# Ammonium Transporter (*BcAMT1.2*) Mediates the Interaction of Ammonium and Nitrate in *Brassica campestris*

**DOI:** 10.3389/fpls.2019.01776

**Published:** 2020-02-04

**Authors:** Yunna Zhu, Xinmin Huang, Yanwei Hao, Wei Su, Houcheng Liu, Guangwen Sun, Riyuan Chen, Shiwei Song

**Affiliations:** ^1^ College of Horticulture, South China Agricultural University, Guangzhou, China; ^2^ College of Yingdong Agricultural Science and Engineering, Shaoguan University, Shaoguan, China

**Keywords:** AMT1.2, *Brassica campestris*, interaction, NH_4_^+^ flux, NO_3_^–^ flux

## Abstract

The provision of ammonium (NH_4_^+^) and nitrate (NO_3_^−^) mixture increases the total nitrogen (N) than the supply of sole NH_4_^+^ or NO_3_^–^ with the same concentration of total N; thus, the mixture contributes to better growth in *Brassica campestris*. However, the underlying mechanisms remain unknown. In this study, we analyzed NH_4_^+^ and NO_3_^–^ fluxes using a scanning ion-selective electrode technique to detect under different N forms and levels in *B. campestris* roots. We observed that the total N influxes with NH_4_^+^ and NO_3_^−^ mixture were 1.25- and 3.53-fold higher than those with either sole NH_4_^+^ or NO_3_^−^. Furthermore, NH_4_^+^ and NO_3_^–^ might interact with each other under coexistence. NO_3_^–^ had a positive effect on net NH_4_^+^ influx, whereas NH_4_^+^ had a negative influence on net NO_3_^–^ influx. The ammonium transporter (AMT) played a key role in NH_4_^+^ absorption and transport. Based on expression analysis, *BcAMT1.2* differed from other *BcAMT1s* in being upregulated by NH_4_^+^ or NO_3_^−^. According to sequence analysis and functional complementation in yeast mutant 31019b, AMT1.2 from *B. campestris* may be a functional AMT. According to the expression pattern of *BcAMT1.2*, β-glucuronidase activity, and the cellular location of its promoter, BcAMT1.2 may be responsible for NH_4_^+^ transport. Following the overexpression of *BcAMT1.2* in *Arabidopsis*, *BcAMT1.2*-overexpressing lines grew better than wildtype lines at low NH_4_^+^ concentration. In the mixture of NH_4_^+^ and NO_3_^–^, NH_4_^+^ influxes and NO_3_^–^ effluxes were induced in *BcAMT1.2*-overexpressing lines. Furthermore, transcripts of N assimilation genes (*AtGLN1.2*, *AtGLN2*, and *AtGLT1*) were significantly upregulated, in particular, *AtGLN1.2* and *AtGLT1* were increased by 2.85–8.88 times in roots, and *AtGLN1.2* and *AtGLN2* were increased by 2.67–4.61 times in leaves. Collectively, these results indicated that *BcAMT1.2* may mediate in NH_4_^+^ fluxes under the coexistence of NH_4_^+^ and NO_3_^–^ in *B. campestris*.

## Introduction

The efficiency and availability of nitrogen (N) have decisive influences on plant growth and crop productivity (Hachiya and Sakakibara, 2017). For most plants, nitrate (NO_3_^–^) and ammonium (NH_4_^+^) are major sources of inorganic N. In C_3_ plants, NO_3_^–^ reduction is inhibited by elevated carbon dioxide (CO_2_), whereas NH_4_^+^ assimilation is affected little (Bloom et al., 2010). NH_4_^+^ is believed to be a preferable N source for the future when global levels of CO_2_ are predicted to increase (Hachiya and Sakakibara, 2017). However, NH_4_^+^ at millimolar concentrations in the soil solution or hydroponic culture causes growth suppression and chlorosis (ammonium toxicity) in plants, unlike NO_3_^–^ at the same concentration (Miller and Cramer, 2004).

Extensive studies suggest that a mixture of NO_3_^–^ and NH_4_^+^ nutrition stimulates plant growth beyond that observed with NO_3_^–^ or NH_4_^+^ alone (Britto and Kronzucker, 2001). The use of the mixture enhances N-use efficiency and improves crop productivity (Wang and Shen, 2011; Hachiya et al., 2012). The mixture greatly improves plant growth and population productivity in maize, especially in high planting density (Wang et al., 2019). When NO_3_^–^ and NH_4_^+^ co-exist, NH_4_^+^ responses are altered by NO_3_^–^ and vice versa (Hachiya and Sakakibara, 2017). Previous researchers have investigated the interaction between NH_4_^+^ and NO_3_^–^ fluxes. Compared with the influx with sole NH_4_^+^, net NH_4_^+^ influx has been shown to increase with a mixture of NH_4_^+^ and NO_3_^–^ in rice using an N labeling technique (Kronzucker et al., 1999); and a similar effect has been observed in *Brassica napus* (Babourina et al., 2007), *Populus popularis* (Luo et al., 2013), and *Triticum aestivum* (Zhong et al., 2015) using the microelectrode technique, whereas a negative effect has been observed in tea (Ruan et al., 2016). Similarly, NH_4_^+^ affects NO_3_^–^ fluxes (Kronzucker et al., 1999; Zhong et al., 2015; Ruan et al., 2016). Therefore, the interaction between NH_4_^+^ and NO_3_^–^ may depend on plant species or N conditions.

Under natural conditions, plant growth and development are typically limited by N availability; thus, plants have evolved different transport and signaling mechanisms to adapt to different N sources (Kiba and Krapp, 2016). NH_4_^+^ and NO_3_^–^ fluxes are mediated by specific genes for ammonium transporters (AMTs) and nitrate transporters (NRTs), respectively (Nacry et al., 2013). In *Arabidopsis*, NRTs include 72 members belonging to four families: nitrate transporter 1/peptide transporter family (NRT1/PTR), NRT2, chloride channels (CLC), and slow anion channel-associated 1/slow anion channel homologs (SLAC1/SLAH) (Krapp et al., 2014). Some of these genes are related to NO_3_^–^ uptake, xylem loading, and efflux systems (Krapp et al., 2014). AMTs generally contain AMT1 and AMT2 subfamilies (Loque and von Wirén, 2004; McDonald and Ward, 2016). In *Arabidopsis*, *AtAMT1.1*, *AtAMT1.2*, *AtAMT1.3*, and *AtAMT1.5* are expressed in roots (Yuan et al., 2007), and play different roles during NH_4_^+^ assimilation (Yuan et al., 2007). *AtAMT1.1*, *AtAMT1.3* and *AtAMT1.5* contribute to NH_4_^+^ absorption from the soil, whereas *AtAMT1.2* mediates NH_4_^+^ uptake *via* the apoplastic transport route (Yuan et al., 2007), and exclusively regulates NH_4_^+^ flux into the vasculature (Straub et al., 2017). Furthermore, plant cells eliminate the activity of AMT1.1 (Lanquar et al., 2009) or AMT1.3 (Wang et al., 2013) to avoid excessive NH_4_^+^ accumulation.

*AMTs* transcript levels are affected by the N status of plants. N deficiency strongly induces *AMT1.1*, *AMT1.3*, and *AMT1.5* transcription (Yuan et al., 2007; Camañes et al., 2009), whereas that of *AMT1.2* is not affected to a large extent (Pearson et al., 2002). When NH_4_^+^ is resupplied to N-deficient plants, *AMT1.1*, *AMT1.3*, and *AMT1.5* genes are downregulated (Yuan et al., 2007); whereas *AMT1.2* is upregulated (Pearson et al., 2002; Yuan et al., 2007). Furthermore, *AMTs* transcript levels are subjected to control by NO_3_^–^ (Camañes et al., 2009). However, AMT homologs in different species are often not similarly regulated, which may reflect the different nutritional needs of particular species (Loque and von Wirén, 2004).

Flowering Chinese cabbage (*Brassica campestris* L. ssp*. chinensis* var. *utilis* Tsen et Lee) is a prominent vegetable in South China due to the taste and nutrient content of its flower stalk, and it has the largest growing area and yield in South China (Song et al., 2012). In our previous study, we showed that NH_4_^+^ and NO_3_^–^ mixtures were more beneficial to *B. campestris* qualities than sole N source, and they improved N-use efficiency (Song et al., 2012). However, there is no information regarding the interactions between NH_4_^+^ and NO_3_^–^ and how this affects N uptake at physiological, morphological, and molecular levels. In this study, we examined the characteristics of NH_4_^+^ and NO_3_^–^ fluxes and their interactions in *B. campestris* using the scanning ion-selective electrode technique (SIET). Regarding the analysis of *AMT1s* transcripts, we observed that the expression pattern of *BcAMT1.2* differed from those of other *BcAMT1s* in *B. campestris*. Furthermore, the GUS activity of *BcAMT1.2_pro_::GUS* and used reverse genetic approaches in *Arabidopsis* suggested to elucidate the physiological roles of *BcAMT1.2* in response to the coexistence of NH_4_^+^ and NO_3_^–^. Altogether, these results indicated that *BcAMT1.2* participated in the interaction between NH_4_^+^ and NO_3_^–^ in *B. campestris*.

## Materials and Methods

### Plant Materials and Culture Conditions

The flowering Chinese cabbage variety “Youlv80”, which was provided by the Guangzhou Academy of Agriculture Sciences (Guangdong Province, China), was used in this study. Experiments were carried out in a controlled-environment growth chamber programmed for 16 h light/8 h dark and a 25/23°C day/night cycle, relative humidity of 70%, and light intensity of 150 μmol m^–2^ s^–1^. Seeds were sterilized in 2.5% (w/v) NaClO for 10 min, washed five times with sterile distilled water, and cultured on vertical 0.7% agar plates (17.5 cm long × 16 cm wide × 3 cm high). The agar medium contained 1/2 no-N basal modified MS salt (pH 5.8), supplemented with 4 mmol L^–1^ NaNO_3_ as the N source. On the 6^th^ day of germination, the seedlings were hydroponically cultured in 1/2 MS as an N-deficient treatment for 7 d. The nutrient solution was replaced every 2 days and continually aerated by air pumps. After N starvation, the seedlings were harvested to measure ion fluxes or other treatments.

### Measurement of NH_4_^+^ and NO_3_^–^ Ion Fluxes on the Surface of *B. campestris* Roots

To monitor net fluxes of NH_4_^+^ and NO_3_^–^ in *B. campestris* roots in response to different N treatments, the primary roots were selected and immersed in measuring solutions with different treatment [A. 0.25 mmol L^–1^ NH_4_^+^: 0.1 mmol L^–1^ CaCl_2_, 0.3 mmol L^–1^ 2-(*N*-morpholino) ethanesulfonic acid hydrate buffer (MES) (pH5.8, same as below), and 0.25 mmol L^–1^ NH_4_Cl; B. 1.0 mmol L^–1^ NH_4_^+^: 0.1 mmol L^–1^ CaCl_2_, 0.3 mmol L^–1^ MES, and 1.0 mmol L^–1^ NH_4_Cl; C. 0.25 mmol L^–1^ NO_3_^–^: 0.1 mmol L^–1^ CaCl_2_, 0.3 mmol L^–1^ MES, and 0.25 mmol L^–1^ NaNO_3_; D. 1.0 mmol L^–1^ NO_3_^–^: 0.1 mmol L^–1^ CaCl_2_, 0.3 mmol L^–1^ MES, and 1.0 mmol L^–1^ NaNO_3;_ E. NH_4_^+^ + NO_3_^–^: 0.1 mmol L^–1^ CaCl_2_, 0.3 mmol L^–1^ MES, 0.25 mmol L^–1^ NH_4_Cl, and 0.75 mmol L^–1^ NaNO_3_]. Prior to analysis, *B. campestris* roots were transferred to Petri dishes containing 10 mL of measuring solution and equilibrated for 10 min. The equilibrated roots were moved to another Petri dish containing fresh measuring solution to measure NH_4_^+^ or NO_3_^–^ flux. Ion flux was measured using SIET (MA01002 system; Younger USA Science and Technology Limited Liability Company, Amherst, MA, USA), which was conducted on-site at Xuyue Science and Technology Company Limited (Beijing, China). The SIET system and its application process for ion flux detection have been previously described in detail (Zhong et al., 2015; Ruan et al., 2016).

To determine the regions along the root where the maximal ion influxes of NH_4_^+^ or NO_3_^–^ occurred, a preliminary experiment was conducted, in which an initial measurement was performed at different points from the root tip (1, 2, 4, 10, 15, 20, 25, 30, and 35 mm). Based on this experiment, we selected 20 and 30 mm from the root apex as the measurement site of NH_4_^+^ and NO_3_^–^ influxes (**Supplementary Figure S2**). The recording rate of ion flux was one reading every 6 s and this lasted for 10 min in each root. Six similar seedlings per treatment were measured.

To evaluate the interaction of NH_4_^+^ and NO_3_^–^ fluxes, the roots of *B. campestris* were soaked in measurement solutions. The effect of NO_3_^–^ on NH_4_^+^ flux [F (with NO_3_^–^): 0.1 mmol L^–1^ CaCl_2_, 0.3 mmol L^–1^ MES, 0.1 mmol L^–1^ NH_4_Cl, and 1 mmol L^–1^ NaNO_3_; G (without NO_3_^–^): 0.1 mmol L^–1^ CaCl_2_, 0.3 mmol L^–1^ MES, 0.1 mmol L^–1^ NH_4_Cl]. The NH_4_^+^ flux was measured using SIET for 3 min after equilibration in measuring solution for 10 min. Thereafter, 1.0 mmol L^–1^ NH_4_Cl was added to the measuring solution, which was mixed thoroughly by expelling and drawing it into a pipette during the first 1–2 min. NO_3_^–^ flux was measured using SIET for 17 min. The effect of NH_4_^+^ on NO_3_^–^ flux [H (with NH_4_^+^): 0.1 mmol L^–1^ CaCl_2_, 0.3 mmol L^–1^ MES, 0.1 mmol L^–1^ NaNO_3_, with 1 mmol L^–1^ NH_4_Cl; I (without NH_4_^+^): 0.1 mmol L^–1^ CaCl_2_, 0.3 mmol L^–1^ MES, 0.1 mmol L^–1^ NaNO_3_]. NO_3_^–^ flux was measured utilizing SIET for 3 min after equilibrated in measurement solution for 10 min. Thereafter, 1.0 mmol L^–1^ NaNO_3_ was added to the measuring solution. The test process was the same as that described above. Six biological replicates were used for each measurement.

### Analysis of *AMTs* and *NRTs* Transcripts in Roots

*B. campestris* seedlings that had been N-starved for 7 d were subjected to different N treatments. The treatments were as follows: (1) exposure to different N levels: 0, 0.25, and 1.0 mmol L^–1^ NaNO_3_/NH_4_Cl were added, then roots were harvested after 20 min during the N-resupply treatments; (2) effect of NH_4_^+^ on NO_3_^–^: 1 mmol L^–1^ NH_4_Cl was added into the solution with or without NaNO_3_, then roots were harvested at 0, 10, and 20 min after adding NH_4_Cl; (3) effect of NO_3_^–^ on NH_4_^+^: 1 mmol L^–1^ NaNO_3_ was added into the solution with or without NH_4_Cl, then roots were harvested at 0, 10, and 20 min after adding NaNO_3_. All samples were immediately frozen in liquid nitrogen and stored at –80°C for quantitative real-time polymerase chain reaction (qPCR).

### qPCR

Total RNA was extracted from samples using an Eastep^®^ Super Total RNA Extraction Kit (Promega, Beijing, China) and was reverse transcribed using a PrimeScript™ RT reagent Kit with gDNA Eraser (TaKaRa Bio, Dalian, China). The qPCR was performed in a LightCycler 480 Real-Time PCR System (Roche, Basel, Switzerland), using SYBR^®^ Premix Ex Taq™ (TaKaRa Bio). The primer pairs used are listed in **Supplementary Table S1**. *GAPDH* was used as an internal control. Three biological replicates were used to calculate relative gene expression levels.

### *BcAMT1.2* Cloning and Sequence Analysis

Based on the *AMT1.2* sequence of *Brassica rapa* (retrieved from GenBank, accessions no. XM_009113156.2), primers (**Supplementary Table S1**) were designed to amplify the full-length of *BcAMT1.2* by PCR using the cDNA of *B. campestris* as the template. The PCR product was cloned into binary vector pCAMBIA3301 (Dingguo Biotechnology, Beijing, China) that carried two CaMV 35S promoters (35S_pro_) and phosphinothricin resistance marker genes and was sequenced. Based on the deduced amino acid sequence, transmembrane motifs, subcellular localization, and signature motifs were predicted using Protter (http://wlab.ethz.ch/protter/), Softberry (http://www.softberry.com), and Weblogo (http://weblogo.berkeley.edu/logo.cgi/), respectively. The multiple sequence alignment of 32 AMT proteins from plants was performed using the ClustalW method and a phylogenetic tree was constructed using MEGA 6.0 based on the neighbor-joining algorithm. Bootstrap analysis was carried out with 1000 replicates. The accession numbers of the amino acid sequences of the AMTs are listed in **Supplementary Table S2**.

### Heterologous Expression of *BcAMT1.2* in Yeast

The open reading frame (ORF) of *BcAMT1.2* was amplified by PCR using the primers (**Supplementary Table S1**) and constructed into pYES2 vector (Waryong Biotechnology, Beijing, China). As described by Yuan et al. (2007), pYES2 and pYES2-BcAMT1.2 plasmids were transformed into yeast mutant cells 31019b (*Δmep1*, *Δmep2*, *Δmep3*, and *ura3*). Growth complementation assays were performed on a solid yeast N base medium at pH 5.8 and were supplemented with 2% galactose and 2 mmol L^–1^ arginine or NH_4_Cl as the sole N source. Yeast cells were incubated at 30°C for 3 days.

### *BcAMT1.2::GUS* Constructs Used for *Arabidopsis* Transformation and β-Glucuronidase (GUS) Assays

The *BcAMT1.2::GUS* construct, containing 1519 bp of *BcAMT1.2* promoter cloned by our lab, was amplified by PCR from the DNA of *B. campestris* using special primers (**Supplementary Table S1**). They were ligated into the pCAMBIA1391 vector which harbored GUS, without a promoter (Dingguo Biotechnology), yielding a pCAMBIA1391-*BcAMT1.2_pro_::GUS* construct. Via *Agrobacterium tumefaciens*-mediated transformation, *BcAMT1.2_pro_::GUS* transgenic plants were generated in a wildtype (Col-0) background. Second generation (T_2_) seeds were germinated on a medium containing 1/2 modified MS, 4 mmol L^–1^ NaNO_3_ and 0.7% agar for 14 d (growth conditions as described above). Some seedlings were subjected to N-free MS treatment for 4 d, and transferred to either the nutrition of N-free MS or the one of N-free MS containing 0.25 mmol L^–1^ NH_4_^+^/NO_3_^–^, and incubated with gentle shaking for 2 h. Histochemical GUS assays were performed as described by Yao et al. (2008). After histochemical staining, seedlings were cleared in 70% ethanol. The images were examined under a digital microscope (VHX-5000; Keyence, Osaka, Japan).

### Generation of BcAMT1.2-Overexpressing *Arabidopsis* Transgenic Lines

Wildtype *Arabidopsis* (Col-0) was transformed with *Agrobacterium* GV3101 harboring the pCAMBIA3301-35S_pro_::*BcAMT1.2* construct. Several transformants were screened by Basta on soil and subjected to PCR analysis using bar primers and qPCR tests of leaves using special *BcAMT1.2* primers (**Supplementary Table S1**). Independent homozygous *BcAMT1.2*-transformed lines were generated in the T_4_ generation.

### Plant Culture for Growth Test, NH_4_^+^ Content, Ion Fluxes, and Gene Expression

For the growth test, surface-sterilized *Arabidopsis* seeds were germinated on a 1/2 MS agar-medium (containing 4 mmol L^–1^ NaNO_3_ as N source) for 4 d and the seedlings were transferred to vertical plates containing 0.25 mmol L^–1^ NH_4_Cl for 10 d. Ten seedlings were used for the measurements of biomass and primary root length. Then, seedlings were mixed to measure the NH_4_^+^ content; 3 biological replicates were used for each line. The measurement of NH_4_^+^ content has been previously described by Ivančič and Degobbis (1984).

For the ion flux test, surface-sterilized *Arabidopsis* seeds were germinated on a 1/2 MS agar-medium (containing 4 mmol L^–1^ NaNO_3_ as N source) for 4 d and transferred to an N-free 1/2 MS agar-medium for 7 d. *Arabidopsis* roots were transferred to a measuring solution (0.1 mmol L^–1^ CaCl_2_, 0.3 mmol L^–1^ MES, 0.25 mmol L^–1^ NH_4_Cl, and 0.75 mmol L^–1^ NaNO_3_) and NO_3_^–^ and NH_4_^+^ fluxes were measured using SIET. Six similar seedlings per treatment were selected to measure ion flux.

*Arabidopsis* seeds were pre-cultured for 4 d (as described above for the ion flux test) and transferred to a 1/2 MS agar-medium (containing 0.25 mmol L^–1^ NH_4_Cl + 0.75 mmol L^–1^ NaNO_3_) for 10 d. Shoots and roots were harvested to isolate total RNA for qPCR analysis and measure the content of NH_4_^+^ and NO_3_^–^, as described by Ivančič and Degobbis (1984) and Downes (1978), respectively. Three biological replicates were used for each measurement. The wildtype was used as control in the above tests.

### Statistical Analysis

Microsoft Excel (Microsoft Corporation, USA) and SPSS 17 (SPSS Incorporation, Chicago, USA) were used to analyze the data. An one-way ANOVA was performed. SigmaPlot 11.1 (Jandel Scientific Software, San Rafael, CA, USA) was utilized to draw figures for data presentation. For gene expression analysis, Hem I software (Heatmap Illustrator, version 1.0) (Deng et al., 2014) was used to generate hierarchical cluster heat maps.

## Results

### Net Fluxes of NO_3_^–^ and NH_4_^+^ in Response to Treatment With Different N Forms and Levels

**Figure 1:**
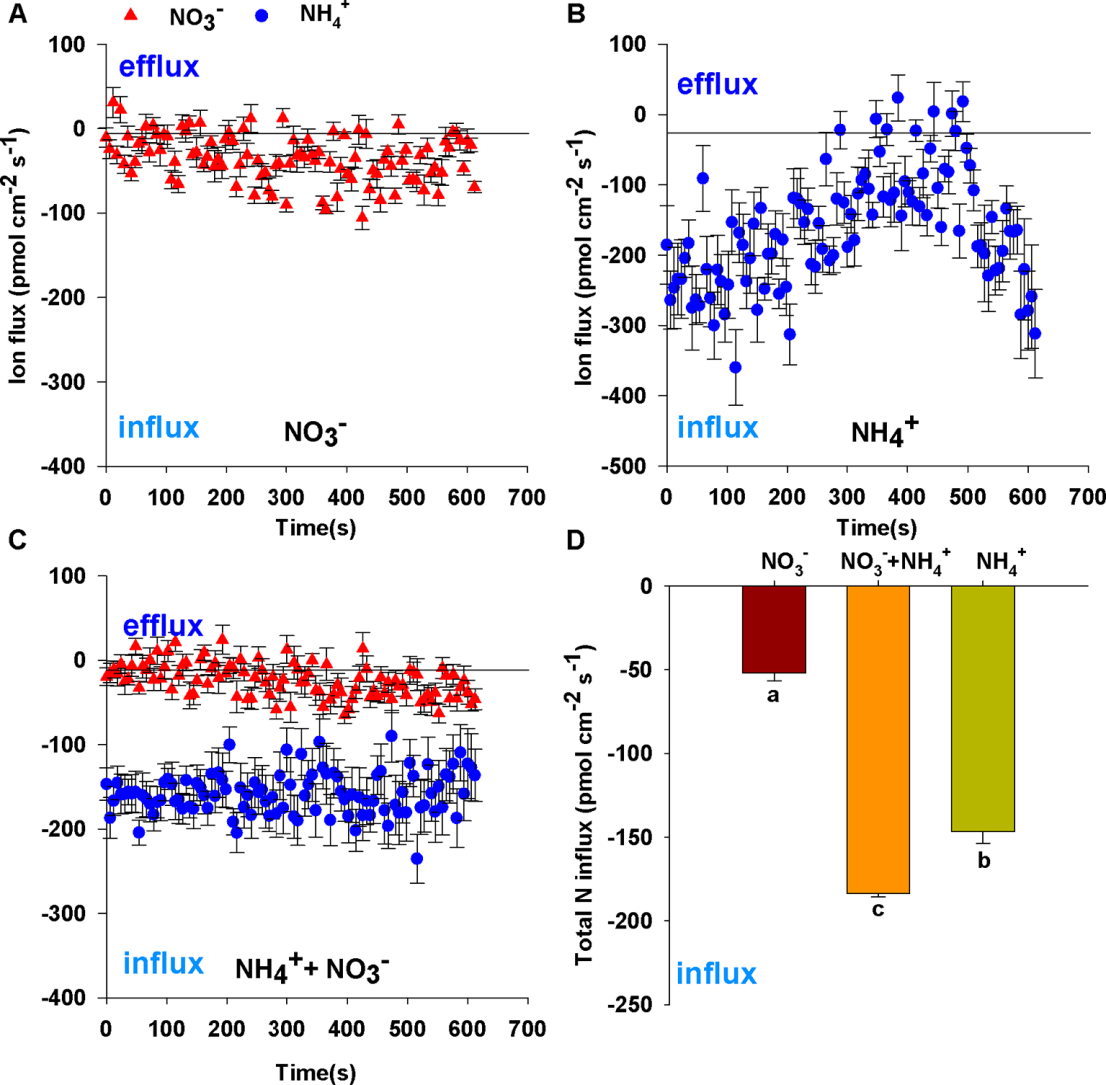
Net fluxes of NO_3_^–^ and NH_4_^+^ on root surfaces of *Brassica campestris* in response to treatments with different N forms. **(A)** Net NO_3_^–^ fluxes under 1 mmol L^–1^ NO_3_^–^; **(B)** net NH_4_^+^ fluxes under 1 mmol L^–1^ NH_4_^+^; **(C)** net NO_3_^–^ and NH_4_^+^ fluxes under mixture of 0.25 mmol L^–1^ NH_4_^+^ and 0.75 mmol L^–1^ NO_3_^–^; **(D)** total N fluxes under different N forms. Net influxes are suggested by negative values, whereas net effluxes are indicated by positive values. The data represent mean ± SE (n = 6). Different letters indicate significant differences at *P <* 0.05.

After 7 d N-starvation, *B. campestris* roots were immersed in measuring solutions containing different N forms (1 mmol L^–1^ NH_4_Cl, 1 mmol L^–1^ NaNO_3_, 0.25 mmol L^–1^ NH_4_Cl + 0.75 mmol L^–1^ NaNO_3_) to monitor net NO_3_^–^ and NH_4_^+^ fluxes. Net NO_3_^–^ and NH_4_^+^ flux curves are shown in **Figures 1A–C**. Net NO_3_^–^ fluxes fluctuated gently in sole NO_3_^–^ (**Figure 1A**) or mixed N (**Figure 1C**). In contrast, net NH_4_^+^ fluxes increased transitorily, then decreased gradually and subsequently increased in sole NH_4_^+^ (**Figure 1B**), whereas net NH_4_^+^ fluxes changed stably in the mixed N treatment (0.25 mmol L^–1^ NH_4_Cl + 0.75 mmol L^–1^ NaNO_3_) (**Figure 1C**). Compared with fluxes in sole N source, NO_3_^–^ fluxes were decreased in mixed N forms and NH_4_^+^ fluxes were close to the fluxes of sole NH_4_^+^ (1 mmol L^–1^ NH_4_Cl) which did not decrease with increasing NH_4_^+^ concentration (**Figures 1A–C**). Thus, the mixed N treatment significantly enhanced total N fluxes (**Figure 1D**) under the same total N conditions (i.e. 3.53-fold for sole NO_3_^–^, 1.25-fold for sole NH_4_^+^).

**Figure 2:**
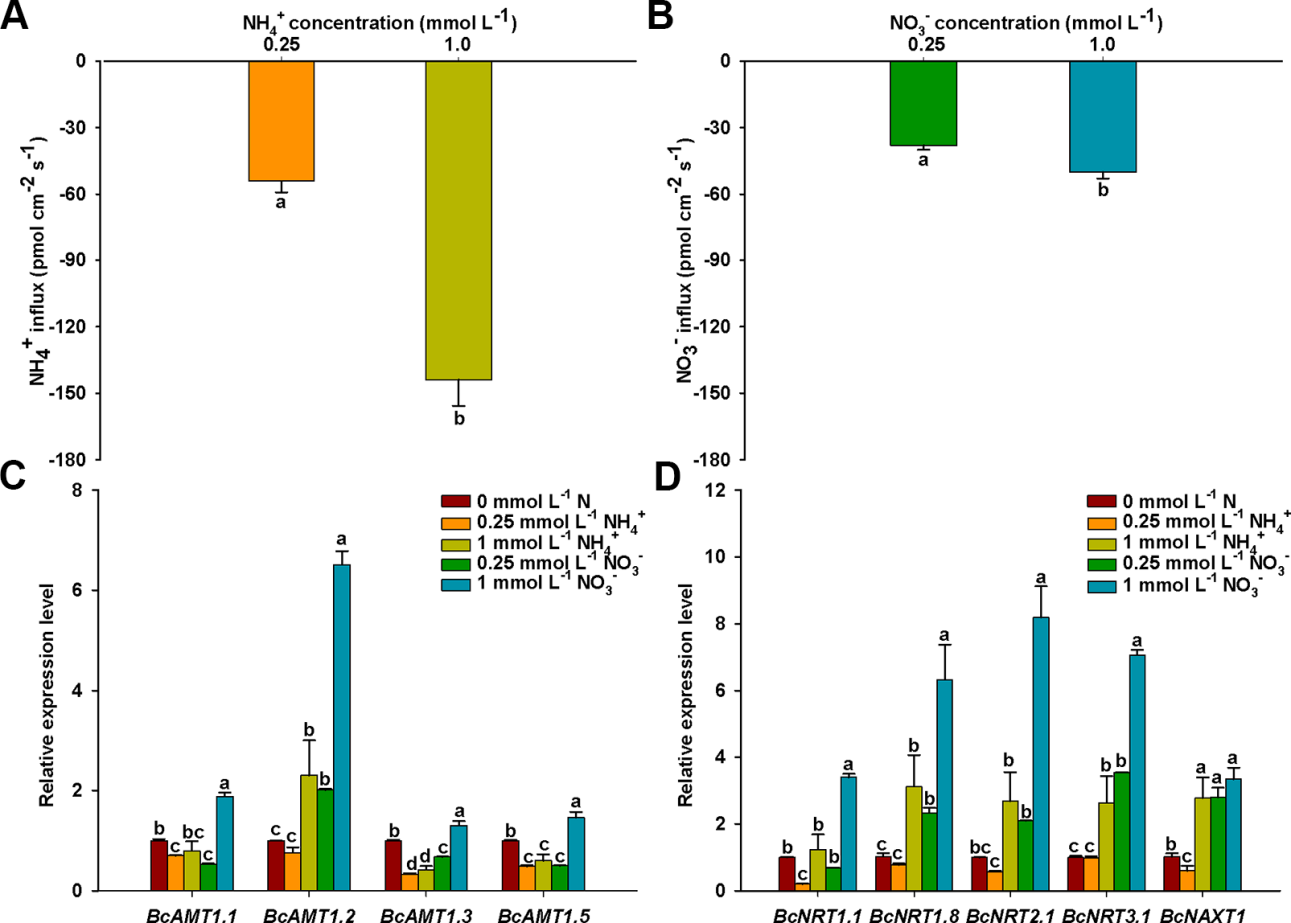
NO_3_^–^ and NH_4_^+^ net fluxes and expressions of *BcAMTs* and *BcNRTs* in *B. campestris* roots in response to treatments with different N levels. **(A)** Net NH_4_^+^ fluxes in different NH_4_^+^ levels (0.25, and 1 mmol L^–1^ NH_4_^+^). **(B)** Net NO_3_^–^ fluxes in different NO_3_^–^ levels (0.25, and 1 mmol L^–1^ NO_3_^–^). **(C, D)** *BcAMTs* and *BcNRTs* expression in different N levels, respectively (0.25, and 1 mmol L^–1^ NH_4_^+^/NO_3_^–^). *GAPDH* was used as internal control. The data represent the mean ± SE (n = 6 in **A**–**B**, n = 3 in **C**–**D**). Significant differences (*P <* 0.05) between treatments are indicated by different letters.

To eliminate the effect of N concentration on N fluxes, we measured that net NO_3_^–^ and NH_4_^+^ fluxes under different N levels. The influx rates of NH_4_^+^ or NO_3_^–^ increased significantly with an increase in N concentration, NH_4_^+^ and NO_3_^–^ influx rates in 1 mmol L^–1^ N were 2.66-fold and 1.33-fold of those in 0.25 mmol L^–1^, respectively (**Figures 2A, B**). In addition, NH_4_^+^ influx rates were 1.42 and 2.88 times higher than those of NO_3_^–^ at N levels of 0.25 and 1 mmol L^–1^, respectively. This indicated that the roots of *B. campestris* showed a preference for NH_4_^+^ over NO_3_^–^.

The absorption of NH_4_^+^ and NO_3_^–^ are mediated by AMTs and NRTs, respectively. To investigate how the expression of the N transporter genes was affected in roots in response to the addition of NH_4_^+^ or NO_3_^–^, we measured the mRNA levels of four *BcAMT* genes (*BcAMT1.1*, *BcAMT1.2*, *BcAMT1.3*, and *BcAMT1.5*) and five *BcNRT* genes (*BcNRT1.1*, *BcNRT1.8*, *BcNRT2.1*, *BcNRT3.1*, and *BcNAXT1*) using qPCR. After a 7-d period of N-starvation, the addition of different N levels had significant effects on the expression levels of *BcAMT* and *BcNRT* genes. Compared with the expression levels at nitrogen starvation (0 mmol L^–1^ N), the expression levels of *BcAMT1.1*, *BcAMT1.3*, and *BcAMT1.5* decreased in response to NH_4_^+^ (0.25 and 1 mmol L^–1^) and 0.25 mmol L^–1^ NO_3_^–^, but they increased in response to 1 mM NO_3_^–^ treatment (i.e. 1.30–1.88 times) (**Figure 2C**). In contrast, *BcAMT1.2* expression increased significantly under 1 mM NH_4_^+^ (i.e. 2.30 times higher), and it was also significantly enhanced with an increase in NO_3_^–^ concentration (i.e. 2.01 and 6.51 times higher in response to 0.25 mmol L^–1^ and 1 mmol L^–1^ NO_3_^–^ treatment, respectively) (**Figure 2C**). *BcAMT1s* expression levels were increased by supplying 1 mmol L^–1^ NO_3_^–^, with the expression of *BcAMT1.1*, *BcAMT1.2*, *BcAMT1.3*, and *BcAMT1.5* being 2.36, 2.83, 3.12, and 2.41 times higher, respectively, than that with the same NH_4_^+^ concentration (**Figure 2C**). In contrast to 0.25 mmol L^–1^ NH_4_^+^, adding NO_3_^–^ enhanced *BcAMT1.2* expression levels (**Supplementary Figure S3A**).

Compared with the expression in nitrogen starvation, *BcNRT1.1* expression was lower following treatment with 0.25 mmol L^–1^ NH_4_^+^, although it did not appear to be affected by treatment with 1 mmol L^–1^ NH_4_^+^. In contrast, although the expression of other *BcNRTs* was not affected by treatment with 0.25 mmol L^–1^ NH_4_^+^, the expression was significantly enhanced in response to treatment with 1 mmol L^–1^ NH_4_^+^ (**Figure 2D**). Except for *BcNRT1.1*, the expression of other *BcNRTs* increased gradually with the concentration of NO_3_^–^ (**Figure 2D**). *BcNRTs* expression was increased by supplying 1 mmol L^–1^ NO_3_^–^, with the expression of *BcNRT1.1*, *BcNRT1.8*, *BcNRT2.1*, *BcNRT3.1,* and *BcNAXT1* being 2.74, 2.03, 3.06, 2.68, and 1.20 times higher than that with the same NH_4_^+^ concentration, respectively (**Figures 2C, D**). In contrast to treatment with 1 mmol L^–1^ NO_3_^–^, adding a mixture of 0.25 mmol L^–1^ NH_4_^+^ and 1 mmol L^–1^ NO_3_^–^ decreased the expression levels of *BcNRT1.8*, *BcNRT2.1*, *BcNRT3.1*, and *BcNAXT1* (**Supplementary Figure S3B**).

### Interactions Between NH_4_^+^ and NO_3_^–^ in Roots of *B. Campestris*

**Figure 3:**
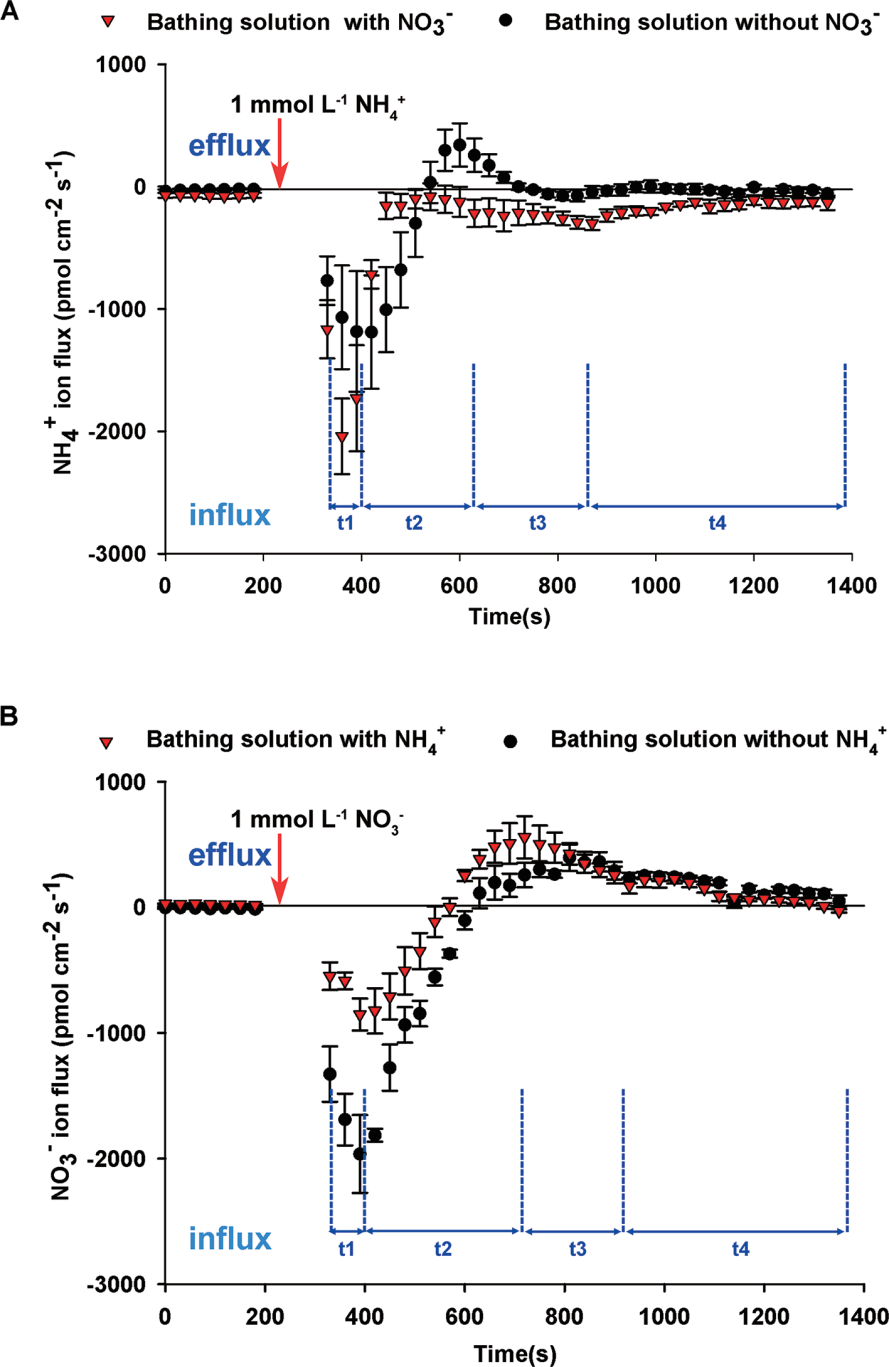
Interaction between NO_3_^–^ and NH_4_^+^ fluxes on root surfaces of *B. campestris*. **(A)** Influence of NO_3_^–^ on net NH_4_^+^ fluxes after adding 1 mmol L^–1^ NH_4_^+^ to the bathing solution with or without 1 mmol L^–1^ NO_3_^–^. **(B)** Influence of NH_4_^+^ on net NO_3_^–^ fluxes after adding 1 mmol L^–1^ NO_3_^–^ to the bathing solution with or without 1 mmol L^–1^ NH_4_^+^. Changes in net NH_4_^+^/NO_3_^–^ fluxes in roots at 30 s intervals are presented. The vertical arrow indicates the point at which 1 mmol L^–1^ NH_4_^+^ or NO_3_^–^ was added. t1–t4 represent the stages of net NH_4_^+^/NO_3_^–^ fluxes after adding NH_4_^+^/NO_3_^–^ to the bathing solution. The data represent the mean ± SE (n = 4–6) during the measurement period.

To elucidate the interaction between NH_4_^+^ and NO_3_^–^, we undertook dynamic monitoring of NH_4_^+^ fluxes after adding NH_4_^+^ to the bathing solution either with or without NO_3_^–^. Before adding NH_4_^+^, net NH_4_^+^ influxes of bathing solution with NO_3_^–^ were higher than that of bathing solution without NO_3_^–^ (**Figure 3A**). Regardless of whether the bathing solution contained NO_3_^–^ or not, net NH_4_^+^ influxes rates after adding NH_4_^+^ increased markedly for 30 to 90 s (t1 stage), then decreased quickly for 180 s (t2 stage), then increased gradually (t3 stage), followed by a slow relaxation to the stable level (t4 stage) (**Figure 3A**). With the exception of several time points in the t2 stage, net NH_4_^+^ influxes of the solution with NO_3_^–^ was higher than that of the solution without NO_3_^–^. There was no obvious difference between NH_4_^+^ flux rates in the bathing solution with or without Na^+^, indicating that adding Na^+^ had no obvious effect on NH_4_^+^ flux in this study (**Supplementary Figure S4**). It indicated that NO_3_^–^ influenced NH_4_^+^ flux rates.

Before adding NO_3_^–^, NO_3_^–^ fluxes of the bathing solution without NH_4_^+^ showed net influxes, whereas those with NH_4_^+^ showed net effluxes (**Figure 3B**). Net NO_3_^–^ influx began to increase rapidly for 60 s (t1 stage) after adding NO_3_^–^ and decreased gradually for 330–420 s (t2 stage). Subsequently, net NO_3_^–^ influx rates increased slowly for approximately 210 s (t3 stage) and remained stable (t4 stage). During the stages t1 and t2, net NO_3_^–^ influx rates of the bathing solution with NH_4_^+^ were lower than those for the bathing solution without NH_4_^+^. There was no obvious difference between the bathing solution with and without NH_4_^+^ during the stages t3 and t4, indicating that NH_4_^+^ affected net NO_3_^–^ influxes.

### *BcAMTs* and *BcNRTs* Expression in Response to Treatment With Adding NH_4_^+^ or NO_3_^–^ in *B. campestris* Roots

**Figure 4:**
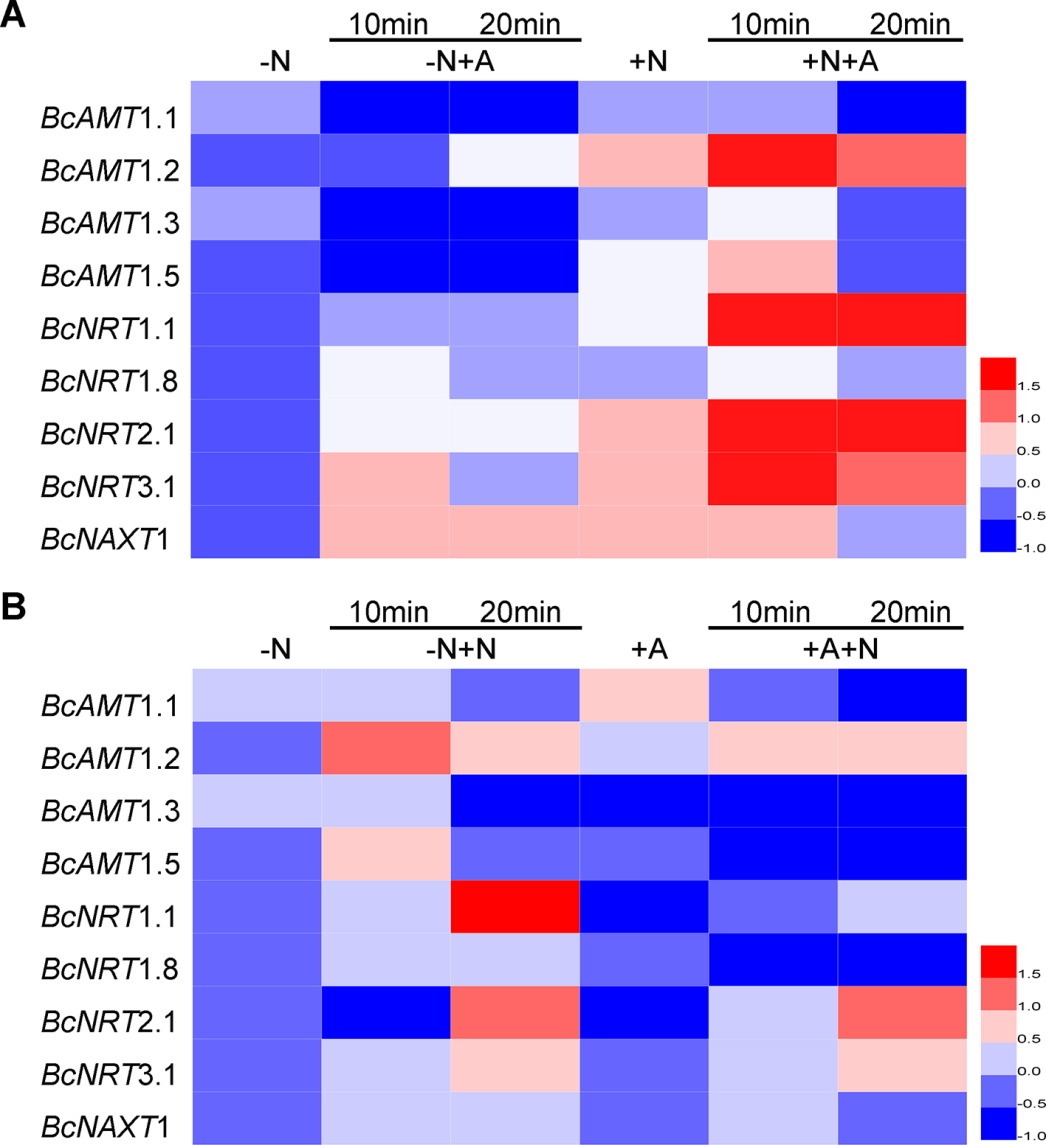
The expression of *AMTs* and *NRTs* in *B. campestris* roots in response to adding NH_4_^+^ or NO_3_^–^ to different N nutrient. **(A)** *AMTs* and *NRTs* expression in seedlings by adding NH_4_^+^ to the nutrient without or with NO_3_^–^. **(B)** *AMTs* and *NRTs* by adding NO_3_^–^ to nutrient solution without or with NH_4_^+^. After 7 d N-starvation, seedlings were transferred to different nutrients. –N: Nitrogen starvation; –N + NH_4_^+^: adding 1 mmol L^–1^ NH_4_^+^ for 10 min and 20 min; +NO_3_^–^: adding 1 mmol L^–1^ NH_4_^+^ for 10 min and 20 min; +NO_3_^–^: adding 1 mmol L^–1^ NO_3_^–^; +NO_3_^–^ + NH_4_^+^: after adding 1 mmol L^–1^ NO_3_^–^ for 10 min, adding 1 mmol L^–1^ NH_4_^+^ for 10 min and 20 min; –N + NO_3_^–^: adding 1 mmol L^–1^ NO_3_^–^ for 10 min and 20 min; +NH_4_^+^: adding 1 mmol L^–1^ NH_4_^+^; +NH_4_^+^ + NO_3_^–^: after adding 1 mmol L^–1^ NH_4_^+^ for 10 min, adding 1 mmol L^–1^ NO_3_^–^ for 10 min and 20 min. *GAPDH* was used as internal control. The heat map represents the values of normalization for different genes.

Compared with the expression in N deficiency, adding NH_4_^+^ without NO_3_^–^ markedly reduced the expression levels of *BcAMT1.1*, *BcAMT1.3,* and *BcAMT1.5*, whereas it induced the expression of *BcAMT1.2* after 20 min (**Figure 4A**). Moreover, adding NH_4_^+^ with NO_3_^–^, resulted in a sharp increase in the expression of *BcAMT1.2* and a weak transient increase in the expression of *BcAMT1.1*, *BcAMT1.3*, and *BcAMT1.5* (**Figure 4A**). Sole NO_3_^–^ treatment increased the expression of *BcAMT1.2* and *BcAMT1.5* and decreased that of *BcAMT1.1* and *BcAMT1.3* (**Figures 4A, B**), whereas adding NO_3_^–^ to the nutrient solution containing NH_4_^+^ resulted in a decrease in the transcript levels of four *BcAMT1s* (**Figure 4B**).

In terms of *NRTs* expression, sole NH_4_^+^ treatment resulted in a slight increase in the expression of *BcNRT1.1*, *BcNRT1.8*, *BcNRT2.1*, and *BcNRT3.1*, and clearly increased the expression of *BcNAXT1* compared with N starvation (**Figure 4A**), whereas sole NO_3_^–^ treatment resulted in a marked increase of five *NRTs* transcripts (**Figure 4B**). However, the effect of adding NO_3_^–^ was more pronounced than that obtained with the combined addition of NO_3_^–^ and NH_4_^+^ (**Figure 4A**). The transcript levels of five *NRTs* were upregulated in response to the addition of NO_3_^–^, whereas *BcNRT1.1*, *BcNRT2.1*, and *BcNRT1.8* expression levels were clearly downregulated by adding NH_4_^+^ and slightly upregulated by the subsequent addition of NO_3_^–^ (**Figure 4B**). However, the expression levels were lower than those obtained in response to the N mixture in which NO_3_^–^ was added for 10 min and NH_4_^+^ was added for another 10–20 min (**Figure 4A**).

Most *BcNRTs* transcripts were induced by NO_3_^–^ and inhibited by NH_4_^+^, whereas *BcAMT1s* transcripts were inhibited by NH_4_^+^ except for *BcAMT1.2*, which was induced by adding NH_4_^+^ and the effect was strengthened by adding NO_3_^–^. Regarding the analysis of *AMT1s* and *NRTs* transcripts, we speculated that *BcAMT1.2* might play an important role in the coexistence of NO_3_^–^ and NH_4_^+^.

### Cloning of a Putative ORF Encoding an AMT1.2 Homolog From *B. campestris*

**Figure 5:**
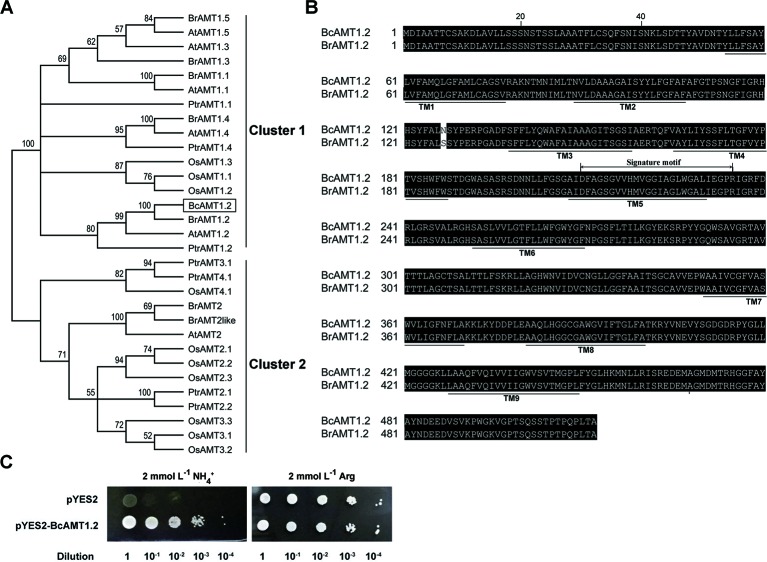
Sequence analysis of BcAMT1.2 and functional complementation in yeast mutant 31019b cells by BcAMT1.2. **(A)** Phylogenetic tree of AMT homologs. It was constructed by the Neighbor-Joining method in MEGA 6.0. Bootstrap values were derived from 1000 replications, and evolutionary distances were estimated in terms of the number of amino acid substitutions per site. The numbers at the nodes are bootstrap values. Accession numbers of protein sequences of AMTs from given plant species are listed in **Supplementary Table S2**. At, *Arabidopsis thaliana*; Bc, *Brassica campestris*; Br, *Brassica rapa*; Os, *Oryza sativa*; Ptr, *Populus trichocarpa*. BcAMT1.2 was represented by a black box. **(B)** Amino acid sequence alignment of AMT1.2 from *B. campestris* and *B. rapa*. The alignment was performed using ClustalW. Amino acids are presented as capital letters; residues are shown in white letters on black if two sequences have identical residues at the aligned positions. Thick lines below sequences show the positions of potential transmembrane α-helices (TMs) as predicted using Protter (http://wlab.ethz.ch/protter/). The sequences marked signature motif indicated a motif specific to the AMT1 sub-family identified using Weblogo (http://weblogo.berkeley.edu/logo.cgi/). **(C)** Functional complementation in yeast mutant 31019b cells by BcAMT1.2. pYES2: empty vector was used as negative control, pYES2-BcAMT1.2: *BcAMT1.2* ORFs was cloned into pYES2 vector. Yeast cell suspensions were adjusted to an optical density at 600 nm of 1.0 (dilution 1), and serially diluted by factors of 10. For each dilution, 3 μL of the yeast cell suspensions were spotted on yeast N base medium with 2 mmol L^–1^ NH_4_^+^ or arginine.

To isolate the *AMT1.2* gene from *B. campestris*, we designed primers based on the sequence of *AMT1.2* from *B. rapa* (accession no. XM_009113156.1) (**Supplementary Table S1**), we obtained the homologous sequence using cDNA from *B. campestris*, designated *BcAMT1.2* (GenBank accession no. MF966937.1). The complete ORF of *BcAMT1.2* consisted of 1539 nucleotides and encoded a 54.94 kD polypeptide. Phylogenetic analysis of AMT1 and AMT2 subfamily members from other plant species showed that BcAMT1.2 belonged to the AMT1 cluster (**Figure 5A**), shared high sequence identity with *Populus trichocarpa* and *Arabidopsis* AMT1.2, and shared 99% identity with *B. rapa* AMT1.2 (**Figure 5B**). It was predicted to be a member protein exhibiting nine transmembrane domains with an N-terminus outside and C-terminus inside the cytoplasm (**Figure 5B**). The sequence of BcAMT1.2 contained the signature motif “^210^DFAGSGVVHMVGGIAGLWGALIEGPR^235^” near the 5^th^ transmembrane domain (**Figure 5B**). The subcellular location in onion cells also showed that *BcAMT1.2* was located in the plasma membrane (**Supplementary Figure S5**).

To investigate whether BcAMT1.2 is a functional ammonium transporter, we recombined the ORF of BcAMT1.2 into pYES2 vector, and transformed this into yeast mutants 31019b. Negative control cells transformed into pYES2 did not grow normally on a solid medium with 2 mmol L^–1^ NH_4_^+^ as the only N source, whereas recombinant strains harboring pYES2-BcAMT1.2 grew normally (**Figure 5C**). This indicated that BcAMT1.2 may be a functional ammonium transporter. *BcAMT1.2* was constitutively expressed throughout the growth period, mainly in roots and leaves, whereas the expression in stems and flowers was lower (**Supplementary Figure S6A**). In roots and leaves, *BcAMT1.2* expression decreased significantly as N starvation progressed (**Supplementary Figures S6B, C**).

**Figure 6:**
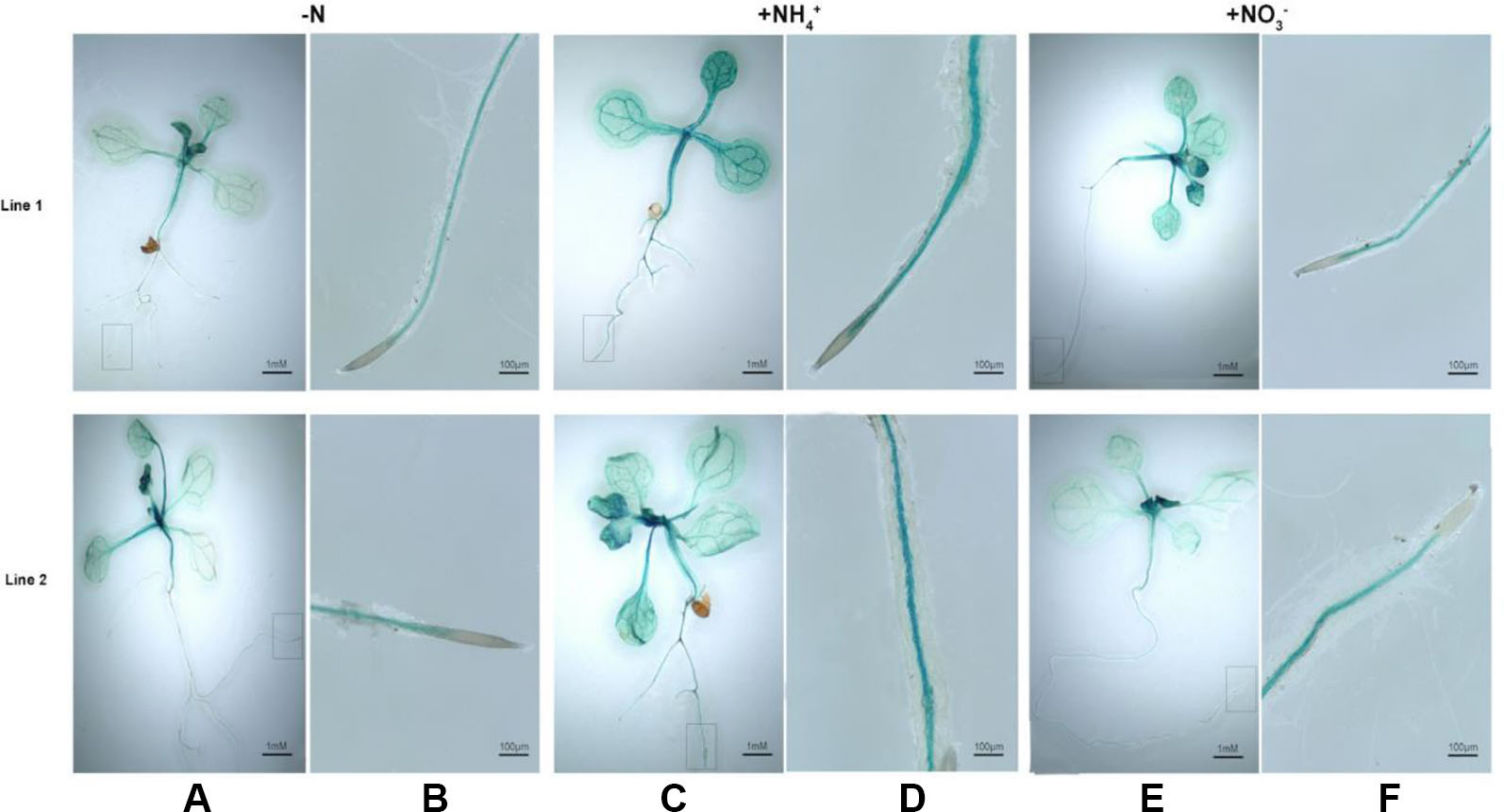
Histochemical staining for GUS activity in *Arabidopsis* seedlings transformed with *BcAMT1.2pro::GUS*. **(A, B)** Seedlings of two lines of transforming with *BcAMT1.2pro::GUS* in *Arabidposis* (Line1, Line2) were subjected to N-deficiency for 4 d. **(C, D)** Seedlings were subjected to 0.25 mmol L^–1^ NH_4_^+^ for 2 h after N-deficiency. **(E, F)** Seedlings were subjected to 0.25 mmol L^–1^ NO_3_^–^ for 2 h after N-deficiency. **(B)**, **(D)**, and **(F)** were the magnification of root zones in a rectangular box of **(A)**, **(C)**, and **(E)**, respectively.

We subsequently investigated that histochemical staining for *BcAMT1.2_pro_::GUS* transformants that were treated with NH_4_^+^, NO_3_^–^, or N-deficiency and stained for GUS activity. In leaves and roots, GUS activity was greater in response to treatment with NH_4_^+^ or NO_3_^–^ (**Figures 6C–F**) than that with N-deficiency (**Figures 6A, B**). GUS was mainly expressed in the vascular tissues of roots and shoots (**Figures 6A–F**). Two lines showed a similar pattern in response to N-deficiency and a low concentration of NH_4_^+^ or NO_3_^–^ after N-deficiency.

### Heterologous Expression of *BcAMT1.2* in *Arabidopsis*

**Figure 7:**
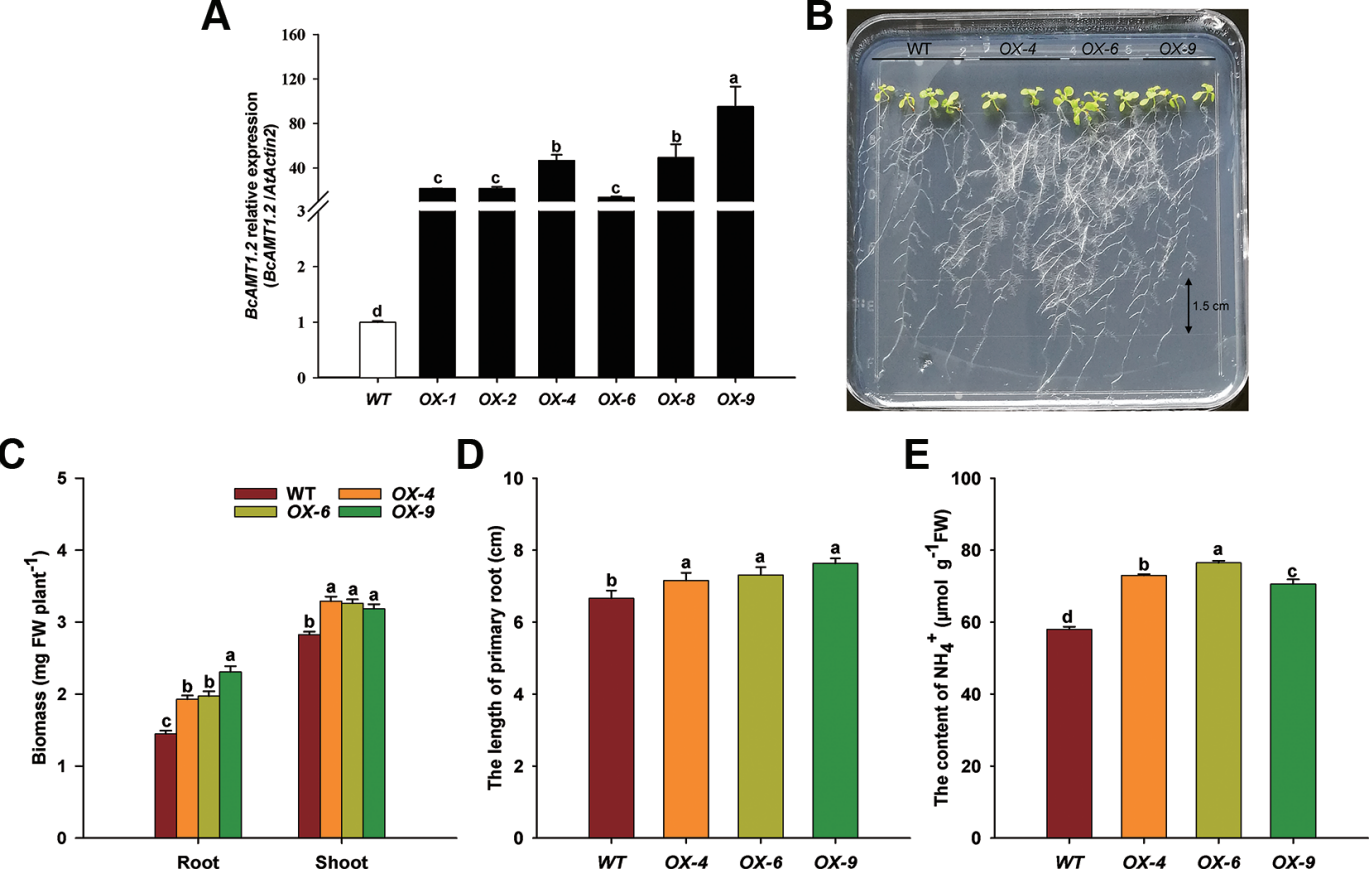
Overexpression of *BcAMT1.2* in *Arabidopsis* promoted plant growth on 0.25 mmol L^–1^ NH_4_^+^. **(A)** Detection of *BcAMT1.2* expression in several transgenic *Arabidopsis* lines. The qPCR was performed on total RNA extracted from the leaves of 2-week-old T_1_ seedlings; *Arabidopsis ACTIN2* was used as internal control. **(B)** Growth phenotype of T_4_ transgenic lines and wildtype on low NH_4_^+^. Seedlings were grown vertically on solid medium containing 0.25 mmol L^–1^ NH_4_^+^ for 10 d after a 4-d pre-culture on 4 mmol L^–1^ NO_3_^–^. Three independent transgenic lines of overexpressed *BcAMT1.2* were used. **(C)** Biomass fresh weight of shoots and roots. **(D)** The length of primary roots. **(E)** NH_4_^+^ content of the whole plants. Each value represents the mean ± SE (n = 3 in **A**, **C** and n = 10 in **D**, **E**). Different lowercase letters indicate significant differences at *P <* 0.05.

To gain an insight into the possible function of *BcAMT1.2* in NH_4_^+^ transportation and utilization in plants, *BcAMT1.2* was overexpressed in the *Arabidopsis* wildtype line (*Col-0*), which was supplied with 0.25 mmol L^–1^ NH_4_^+^ as the sole N source. Several independent homozygous lines harboring *BcAMT1.2* were constructed and the expression of *BcAMT1.2* in *Arabidopsis* was confirmed by qPCR (**Figure 7A**). These seedlings were grown for 10 d on vertical agar plates containing 0.25 mmol L^–1^ NH_4_Cl after a 4-d pre-culture on 4 mmol L^–1^ NaNO_3_. The growth phenotype of transgenic lines showed that the overexpression of *BcAMT1.2* could promote the growth of *Arabidopsis* seedlings at a low concentration of NH_4_^+^ (**Figure 7B**). Compared with the biomass in the wildtype, three *BcAMT1.2*-overexpressing (*BcAMT1.2-ox*) lines significantly increased the biomass of shoots and roots (**Figure 7C**), and the length of primary root (**Figure 7D**). Furthermore, NH_4_^+^ content was increased by 17.9–32.0% in *BcAMT1.2-ox* lines (**Figure 7E**).

### Ion Fluxes of Overexpression *BcAMT1.2* Lines in *Arabidopsis* Under Coexistence of NH_4_^+^ and NO_3_^–^

**Figure 8:**
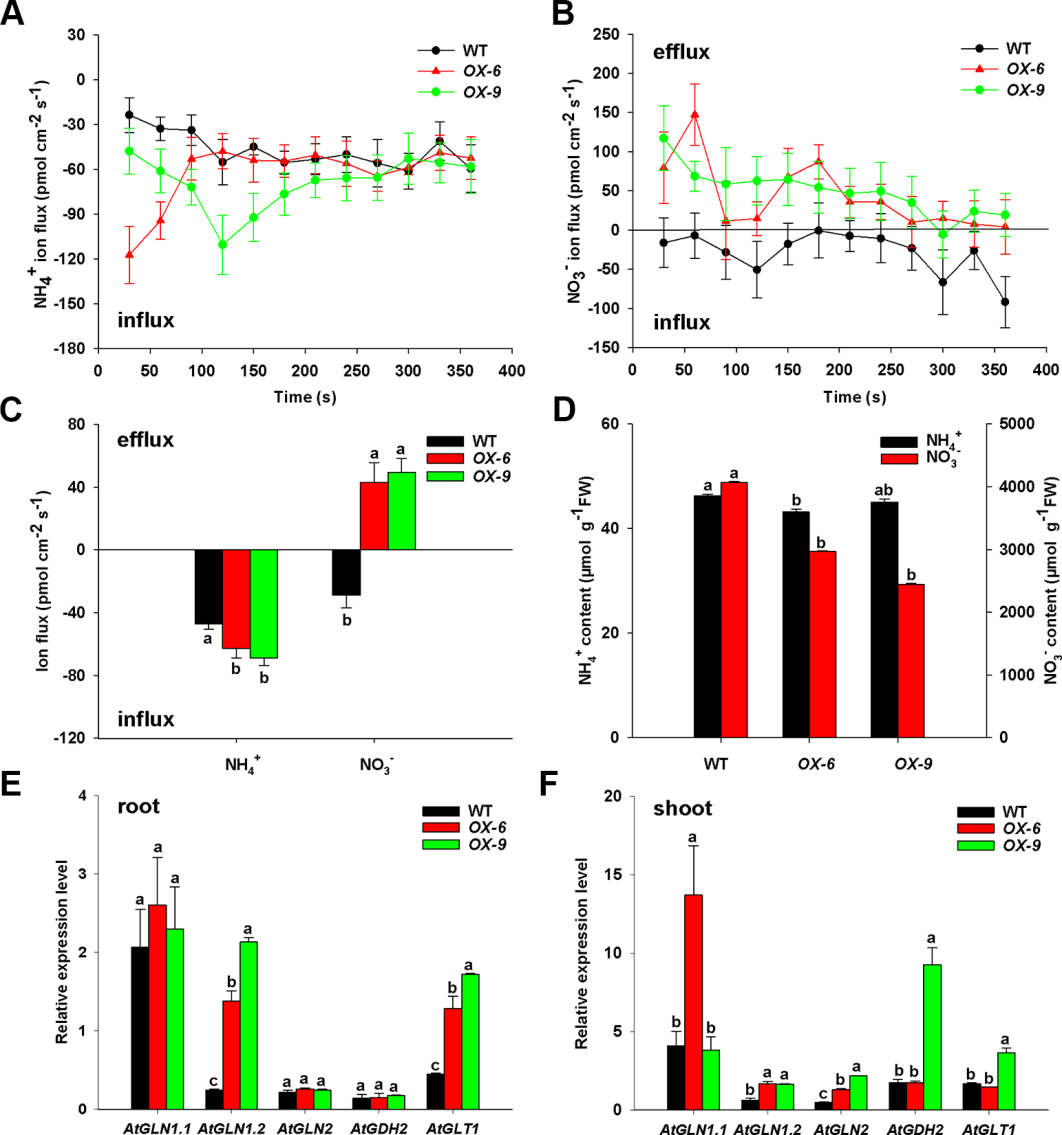
Net ion fluxes and content of NH_4_^+^, NO_3_^–^, and expression of N assimilation genes in *Arabidopsis* wildtype and *BcAMT1.2-ox* lines (*OX-6*, *OX-9*) under a mixture of 0.25 mmol L^–1^ NH_4_^+^ and 0.75 mmol L^–1^ NO_3_^–^. **(A, B)** Net fluxes NH_4_^+^ and NO_3_^–^ of wildtype and *BcAMT1.2-ox* lines. **(C)** Mean values of NH_4_^+^ and NO_3_^–^ net fluxes during the whole test time from **(A)** and **(B)**. **(D)** The content of NH_4_^+^ and NO_3_^–^ in wildtype and *BcAMT1.2-ox* lines. **(E, F)** The expression levels of N assimilation genes in roots and leaves, respectively. *Arabidopsis ACTIN2* was used as internal control. Each value represents the mean ± SE (n = 6 in A–D, n = 3 in **E–F**). Different lowercase letters indicate significant differences at *P <* 0.05.

To examine how *BcAMT1.2-ox* lines affected the absorption of NH_4_^+^ and NO_3_^–^, we measured ion flux rates of *Arabidopsis* seedlings in response to the mixture of N (0.25 mmol L^–1^ NH_4_^+^ and 0.75 mmol L^–1^ NO_3_^–^) using SIET. *BcAMT1.2-ox* lines *OX-6* and *OX-9* showed larger net NH_4_^+^ influxes than the wildtype, and but had little difference in the last minutes of the experiment (**Figure 8A**). *BcAMT1.2-ox* lines influenced NO_3_^–^ flux, which was changed significantly from net influxes to net effluxes in the *BcAMT1.2-ox* line (**Figure 8B**). During the test process, *BcAMT1.2-ox* lines increased 32.8–45.7% in net NH_4_^+^ influx and 2.50–2.72-fold in net NO_3_^–^ efflux in response to a mixture of NH_4_^+^ and NO_3_^–^ (**Figures 8A–C**). These observations indicated that overexpression of *BcAMT1.2* increased NH_4_^+^ influxes and NO_3_^–^ effluxes in *Arabidopsis*. The results of NO_3_^–^ content showed a similar tendency (**Figure 8D**); however, *BcAMT1.2-ox* lines had little influence on NH_4_^+^ content and even reduced it (**Figure 8D**).

To understand if the overexpression of *BcAMT1.2* will affect N assimilation, we investigated the expression levels of five N assimilation genes in *Arabidopsis* under a mixture of NH_4_^+^ and NO_3_^–^. *GLN*, *GDH* and *GLT* encode glutamine synthetase (GS), glutamate dehydrogenase (GDH), and NADH-dependent glutamate synthase (GOGAT), respectively. In roots, the transcript levels of *AtGLN1.2* and *AtGLT1* were 5.73–8.88-fold and 2.85–3.83-fold higher in *BcAMT1.2-ox* lines than those in the wildtype (**Figure 8E**), respectively; in leaves, *AtGLN1.2* and *AtGLN2* transcript levels were 2.67–2.76-fold and 2.71–4.61-fold higher in both *BcAMT1.2*-*ox* lines than those in the wildtype, respectively (**Figure 8F**). Other genes were affected little, either significantly or inconsistently, between two *BcAMT1.2-ox* lines (**Figures 8E, F**). Elevated transcription of N assimilation genes (i.e. *GLN1.2*, *GLN2*, and *GLT1*) might be physiologically crucial for the plants to effectively assimilate and utilize the higher levels of NH_4_^+^ induced by overexpressing *BcAMT1.2*, to retain NH_4_^+^ at a relatively stable level.

## Discussion

### Characteristics of NH_4_^+^, NO_3_^–^ Fluxes, and Related Genes Expression in the Roots of *B. Campestris*

Compared with the growth with a sole N source, a mixture of NO_3_^–^ and NH_4_^+^ accelerates plant growth (**Supplementary Figure S1**) (Wang and Shen, 2011; Song et al., 2012). Plants often show a preference for the uptake of NH_4_^+^ or NO_3_^–^ (Song et al., 2016). Previous studies have shown that molecule-specific activities associated with net NO_3_^–^ and NH_4_^+^ fluxes can be evaluated non-invasively using SIET (Xu et al., 2006). In this study, we observed that the total N influx of the NH_4_^+^ and NO_3_^–^ mixture was higher than that of sole NH_4_^+^ or NO_3_^–^ at the same N amount (**Figures 1A–D**), which is consistent with previous studies on wheat (Zhong et al., 2015) and tea (Ruan et al., 2016). However, it is contrary to the results reported by Arkon et al. (2012), who show a significant decrease of total N uptake in *B. napus* by an NH_4_^+^ and NO_3_^–^ mixture. NH_4_^+^ or NO_3_^–^ uptake is affected by the depolarization of electrical membrane potential which increases with the increase in NH_4_^+^ or NO_3_^–^ concentration, reaches the peak and changes to be steady, according to the Michaelis-Menten equation (Wang et al., 1994). We observed similar results in **Figures 2A, B**. However, at the same concentration, the net influx of NH_4_^+^ was greater than that of NO_3_^–^ in the roots of *B. campestris* (**Figures 1A–D**; **Figures 2A, B**), and at the concentrations of 0.25 mmol L^–1^ and 1 mmol L^–1^, net NH_4_^+^ uptake was 1.42-fold and 2.88-fold higher than net NO_3_^–^ uptake, respectively (**Figures 2A, B**). This indicated that *B. campestris* exhibited a preference for NH_4_^+^ over NO_3_^–^. Previous studies have made similar observations (Zhong et al., 2015; Ruan et al., 2016). Indeed, many plants use NH_4_^+^ as their preferred N form (Socci and Templer, 2011) and most plants prefer to absorb NH_4_^+^ rather than NO_3_^–^ when NH_4_^+^ and NO_3_^–^ are supplied at the same concentration (Zhong et al., 2015; Ruan et al., 2016). Arkon et al. (2012) reported that N uptake and plant growth in *B. napus* are no significantly affected by adding NH_4_^+^ or mixed N during the first 24**–**72 h, whereas causes N uptake and plant growth to decrease after 15 days of treatment compared with NO_3_^–^ treatment. This may be associated with ammonium toxicity (Arkon et al., 2012; Hachiya et al., 2012; Hachiya and Sakakibara, 2017). Therefore, *B. campestris* plant prefers NH_4_^+^ to NO_3_^–^ on the premise that ammonium toxicity cannot affect plant cells in a short time.

In plants, the absorption of NH_4_^+^ or NO_3_^–^ is mainly regulated by *AMT* or *NRT* genes, respectively (Glass et al., 2002), and their expression levels are regulated by N status and forms (Gazzarrini et al., 1999; Yuan et al., 2007). In this study, compared with the transcripts in N-deficiency, *BcAMT1.1*, *BcAMT1.3*, and *BcAMT1.5* transcripts were repressed by adding NH_4_^+^ and affected slightly by NO_3_^–^, whereas *BcAMT1.2* expression was induced by both NH_4_^+^ and NO_3_^–^ (**Figure 2C**). The response of *BcAMT1.1*, *BcAMT1.3*, and *BcAMT1.5* to NH_4_^+^ was similar to the results in *Arabidopsis* (Gazzarrini et al., 1999; Yuan et al., 2007). Those of *BcAMT1.2* to NH_4_^+^ and NO_3_^–^ were consistent with previous results (Pearson et al., 2002; Yusuf and Deepa, 2017). *BcNRTs* transcripts were more affected by NO_3_^–^ than NH_4_^+^, as they were upregulated with an increase in NO_3_^–^ concentration (**Figure 2D**). This is consistent with previous studies (Fan et al., 2016; Qu et al., 2016). Consequently, we conclude that N status and form influence *AMT* and *NRT* transcripts and that these genes are involved in the regulation of NH_4_^+^ and NO_3_^–^ fluxes, respectively.

### NO_3_^–^ Accelerates Net NH_4_^+^ Influxes in *B. campestris*

Previous studies have reported that NH_4_^+^ and NO_3_^–^ might interact with each other under coexistence (Hachiya et al., 2012). Net N fluxes include total N influxes and total N effluxes. When net N influxes increased, total N influxes were enhanced, and/or total N effluxes were reduced (Hachiya and Sakakibara, 2017). In this study, net NH_4_^+^ influxes, with and without containing NO_3_^–^, increased sharply, then decreased rapidly, and slowly relaxed to a stable level with the addition of NH_4_^+^ (**Figure 3A**). Drastic initial changes in NH_4_^+^ fluxes may be caused by depolarization and polarization which are affected by electrical membrane potential after adding more NH_4_^+^ (Wang et al., 1994).

In addition, at a high external concentration of NH_4_^+^, plants may activate the NH_4_^+^ efflux system to cope with high NH_4_^+^ influx (Britto and Kronzucker, 2001; Babourina et al., 2007; Hachiya and Sakakibara, 2017). However, to date there have been no reports of any gene that encodes protein that is specifically involved in the NH_4_^+^ efflux system (Babourina et al., 2007), NH_4_^+^ effluxes may be mediated *via* aquaporin channels or non-selective K^+^ channels (Hachiya and Sakakibara, 2017). Babourina et al. (2007) reported that K^+^ net fluxes are not correlated with net NH_4_^+^ fluxes. Moreover, before adding NH_4_^+^, net NH_4_^+^ influxes in bathing solution containing NO_3_^–^ were higher than in those lacking NO_3_^–^. A similar tendency was observed after adding NH_4_^+^ (**Figure 3A**). This indicated that the presence of NO_3_^–^ might have a positive effect on net NH_4_^+^ uptake, which is consistent with previous studies performed on other species (Kronzucker et al., 1999; Babourina et al., 2007; Luo et al., 2013); however, it is contrary to the results reported by Arkon et al., 2012. Using isotope labeling, Kronzucker et al. (1999) reported that a larger proportion of ^13^NH_4_^+^ signal is allocated to the xylem in the presence of both NH_4_^+^ and NO_3_^–^ than that with sole NH_4_^+^. NO_3_^–^ may influence the expression of *AMTs* involved in cytosolic NH_4_^+^ homeostasis or be involved in a more complex feedback response *via* plant metabolism (Babourina et al., 2007; Hachiya and Sakakibara, 2017). In *Arabidopsis*, NO_3_^–^ mediates NH_4_^+^ uptake and assimilation by *NRT1.1* (Jian et al., 2018).

Compared with the transcript levels in N-deficiency, *BcAMT1.1*, *BcAMT1.3*, and *BcAMT1.5* transcript levels were repressed by NH_4_^+^ to the growth medium, whereas levels were unaffected or increased slightly in response to NO_3_^–^ (**Figures 4A, B**). Nevertheless, *BcAMT1.2* expression was significantly reduced by N-deficiency (**Supplementary Figures S6B, C**), and enhanced by the addition of NH_4_^+^, particularly in the presence of NO_3_^–^ (**Figures 4A, B**). The increased AMT activity may lead to a higher rate of NH_4_^+^ uptake into internal compartments (vacuole or plastids) or further transport to the xylem. Both events would lead to a lower NH_4_^+^ concentration in the cytoplasm of root cells (Babourina et al., 2007). In *Arabidopsis*, AtAMT1.1, AtAMT1.3, and AtAMT1.5 are located in rhizodermal cells, and AtAMT1.2 is located in root endodermal and cortical cells (Yuan et al., 2007). Specific localization in the root zone of AMTs determines the pathways of NH_4_^+^ uptake, transport and allocation to shoots (Duan et al., 2018). When external NH_4_^+^ is high, apoplastic transport mediated by AtAMT1.2 prevails at the root endodermis (Yuan et al., 2007; Duan et al., 2018). AtAMT1.2 exclusively regulates NH_4_^+^ flux into the vasculature (Yuan et al., 2007; Straub et al., 2017) and favors N allocation to the shoot (Duan et al., 2018). *BcAMT1.2_pro_::GUS* activity, which was expressed mainly in the vascular tissues in *Arabidopsis*, was enhanced by adding NH_4_^+^ or NO_3_^–^ compared with that in N-deficiency (**Figures 6A–F**). Therefore, we speculated that *BcAMT1.2* may participate in the interaction of NH_4_^+^ and NO_3_^–^.

### NH_4_^+^ Decreases Net NO_3_^–^ Influxes in *B. campestris*

NH_4_^+^ had an influence on NO_3_^–^ fluxes. Before and after adding NO_3_^–^, net NO_3_^–^ influxes of bathing solution containing NH_4_^+^ were lower than those without NO_3_^–^, whereas net NO_3_^–^ effluxes of bathing solution with NH_4_^+^ were lower than those without NH_4_^+^ (**Figure 3B**). This indicated that NH_4_^+^ might decrease net NO_3_^–^ influxes, which is consistent with the discoveries in other plants (Kronzucker et al., 1999; Arkon et al., 2012; Luo et al., 2013). *BcNRT1.1* and *BcNRT2.1*, a dual-affinity transport system and high affinity transport system, respectively, were downregulated by NH_4_^+^ (**Figures 4A, B**). Furthermore, the expression of *BcNRT1.8*, which regulates the xylem loading of NO_3_^–^, was decreased by NH_4_^+^, whereas that of *BcNAXT1*, which regulates NO_3_^–^ efflux system, was increased (**Figures 4A, B**). The addition of NH_4_^+^ not only decreased NO_3_^–^ absorption, but also NO_3_^–^ xylem loading, and consequently NO_3_^–^ influxes were decreased or NO_3_^–^ effluxes were increased. Previous studies have reported that the acidification of the rhizosphere caused by NAXT1 inhibits NO_3_^–^ absorption (Hachiya and Sakakibara, 2017). Furthermore, the overexpression of *OsNRT2.3b* enhances NO_3_^–^ uptake in response to sole NO_3_^–^ treatment, whereas *OsNRT2.3b* expression is inhibited in response to treatment with mixtures of NH_4_^+^ and NO_3_^–^ (Fan et al., 2016). Therefore, NH_4_^+^ may affect the absorption of NO_3_^–^ by regulating *NRT* transcripts in the coexistence of NH_4_^+^ and NO_3_^–^.

### *BcAMT1.2* Mediated the Interaction of NH_4_^+^ and NO_3_^–^ Coexistence

One AMT1-type homologous gene, namely *BcAMT1.2*, was isolated from *B. campestris* (**Figures 5A, B**). The protein encoded by *BcAMT1.2*, which is located in the plasma membrane, may be a functional AMT (**Supplementary Figure S4**). In a low concentration of NH_4_^+^, overexpressing *BcAMT1.2* lines accelerated the growth of *Arabidopsis* which increased NH_4_^+^ content compared with the wildtype (**Figures 7B–E**). This is consistent with overexpressing *AtAMT1.2* in *Arabidopsis* mutant lines (Yuan et al., 2007). In the NH_4_^+^ and NO_3_^–^ mixture, net NH_4_^+^ influxes of *BcAMT1.2*-ox lines were obviously increased (**Figure 8A**), and net NO_3_^–^ influxes were decreased and changed from net influxes to net effluxes (**Figure 8B**), NO_3_^–^ content of *BcAMT1.2-ox* lines was lower than that of the wildtype (**Figure 8D**), indicating that the constitutive expression of *BcAMT1.2* clearly reduced the NO_3_^–^ influx into roots. Although net NH_4_^+^ influxes of *BcAMT1.2*-*ox* line were increased (**Figures 8A, C**), NH_4_^+^ content was not increased, in contrast to the wildtype (**Figure 8D**).

The expression of N assimilation genes is regulated by NH_4_^+^ in plants (Ranathunge et al., 2014). Previous studies have shown that GS and GOGAT can remove NH_4_^+^ from the cytoplasm to relieve its toxicity (Babourina et al., 2007; Hachiya and Sakakibara, 2017). In *Arabidopsis*, *GLN1* and *GLN2* encode GS isoenzymes, located in the cytosol (GS1) and chloroplast (GS2), respectively (Lothier et al., 2011; Guan et al., 2016). *GLN1.2* in *Arabidopsis* is essential for NH_4_^+^ detoxification and N assimilation under ample nitrate supply (Lothier et al., 2011). GOGAT, encoded by *GLT1*, is responsible for NH_4_^+^ assimilation in non-photorespiratory organs with GDH (Liu and von Wirén, 2017). We observed that the expression levels of N assimilation genes (*AtGLN1.2*, *AtGLN2*, and *AtGLT1*) were significantly increased (**Figures 8E, F**), implying that an increase in *BcAMT1.2* mRNA abundance could also directly or indirectly affect NH_4_^+^ assimilation. *SaAMT1.2* expression levels have been observed to be positively correlated with GS-specific activity in sandalwood (*Santalum album*) (Yusuf and Deepa, 2017). Overexpressing *OsAMT1.1* in rice increases the amounts of amino acids, photosynthetic pigments, and sugars with higher NH_4_^+^ levels to improve nitrogen use efficiency, plant growth, and grain yield (Ranathunge et al., 2014). Therefore, overexpressing *BcAMT1.2* may affect the homeostasis between nitrogen and carbon to regulate plant growth.

However, the mechanisms underlying this phenomenon remain unknown; thus, warrant further investigation in the future. Co-expression experiments in oocytes have revealed that a complex of AMT1.2 with CBL1 and the active CIPK23 kinase is required for AMT1.2 regulation, whereas a noncatalytic CIPK23 is not sufficient to inactivate NH_4_^+^ transportation. CIPK23 and CBL1 appear to occupy a key position in cellular NH_4_^+^ and NO_3_^–^ homeostasis (Straub et al., 2017). Interestingly, the phosphorylation of NRT1.1 is also regulated by CIPK23 (Ho and Tsay, 2010).

In addition, Giehl et al. (2017) reported that *AtAMT2.1* contributes to NH_4_^+^ uptake in the millimolar range, and mediates a high accumulation of NH_4_^+^ in xylem sap, which contributes to long-distance translocation from root to shoot. Further studies are required to clarify whether *BcAMT2s* play a similar role in the interaction between NH_4_^+^ and NO_3_^–^ in *B. campestris*. It may be related to NO_3_^–^ signaling, uptake, and reduction during the interaction of NH_4_^+^ and NO_3_^–^ (Hachiya et al., 2012). How AMT1.2 affects the interaction between NH_4_^+^ and NO_3_^–^ to exert its effects, and whether other proteins and signaling cascades are involved, are interesting questions that await future research.

## Data Availability Statement

All datasets for this study are included in the article/**Supplementary Material**.

## Author Contributions

SS and RC conceived and designed the research. YZ and XH carried out the experiments. WS analyzed the data. YH, GS, and HL reviewed and edited the manuscript.

## Funding

This work was supported by the National Natural Science Foundation of China (31972481, 31401855) and the China Agriculture Research System (CARS-25-C-04).

## Conflict of Interest

The authors declare that the research was conducted in the absence of any commercial or financial relationships that could be construed as a potential conflict of interest.

## Abbreviations:

AMT, ammonium transporter; CBL, calcineurin B-like protein; CIPK, CBL-interacting serine/threonine-protein kinase; GAPDH, glyceraldehyde-3-phosphate dehydrogenase; GDH, glutamate dehydrogenase; GOGAT, glutamate dehydrogenase and NADH-dependent glutamate synthase; GS, glutamine synthetase; GUS, β-glucuronidase; HATS, high-affinity transport system; KD, kilo-dalton; MES, 2-(*N*-morpholino) ethanesulfonic acid hydrate buffer; LATS, low-affinity transport system; N, nitrogen; NH_4_^+^, ammonium; NO_3_^–^, nitrate; NRT, nitrate transporter; ORF, open reading frame; qPCR, quantitative real-time polymerase chain reaction; SIET, scanning ion-selective electrode technique; TM, transport membrane.
