# Supplementary material for: Ammonium Transporter (BcAMT1.2) Mediates the Interaction of Ammonium and Nitrate in Brassica campestris
Source: Front Plant Sci. 2020 Feb 4;10:1776. doi: 10.3389/fpls.2019.01776 (PMC7011105; doi:10.3389/fpls.2019.01776)
Supplement: Supplementary file 1 [file DataSheet_1.docx]

**Title**

**A****mmonium Transporter (*BcAMT1.2*) Mediates the Interaction of Ammonium and Nitrate in *Brassica campestis***

**Supplementary Data**


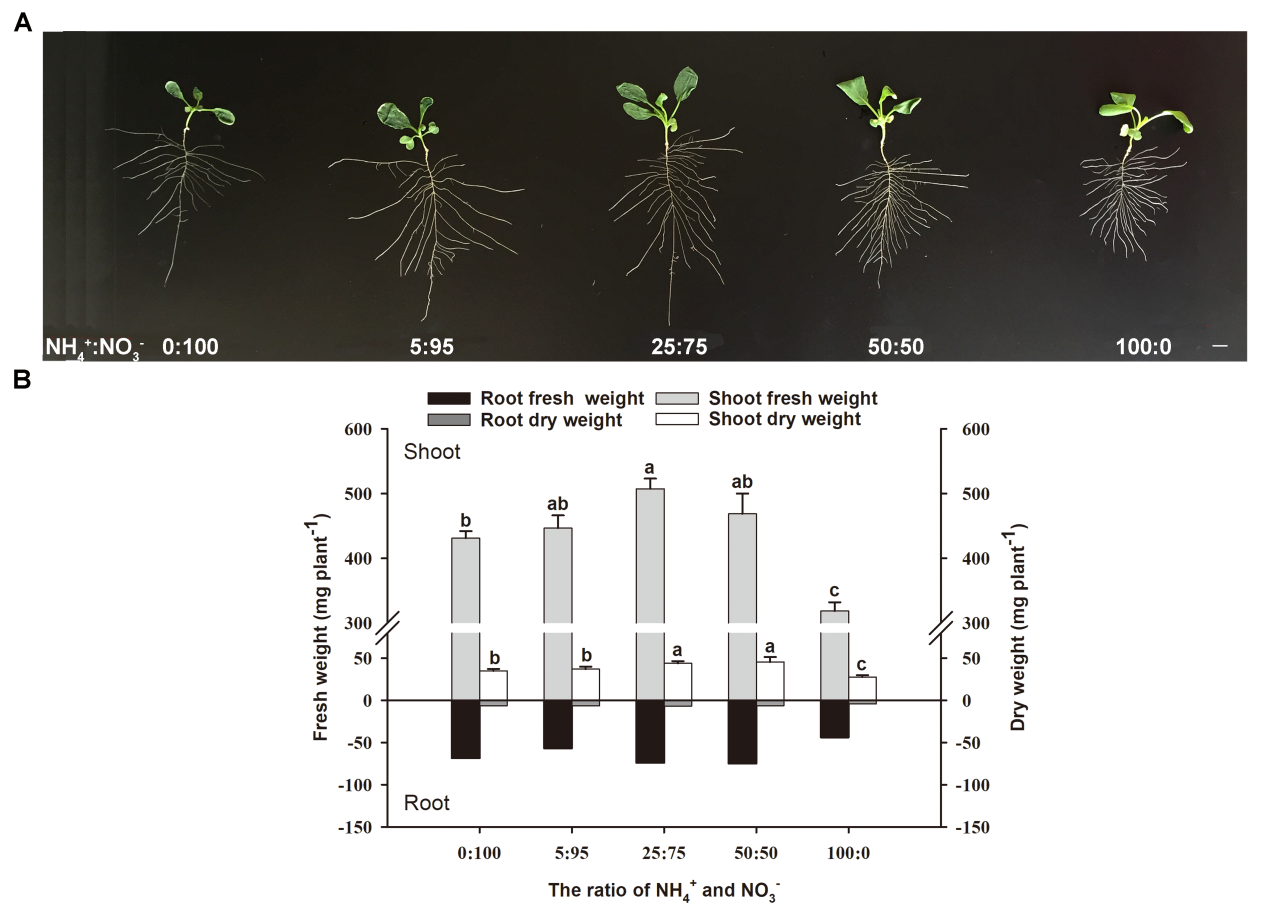


**Supplementary Figure S1 |** Effect of different NH_4_^+^ and NO_3_^-^ ratios on the growth **(A)** and biomass **(B)** of *B. campestris*.


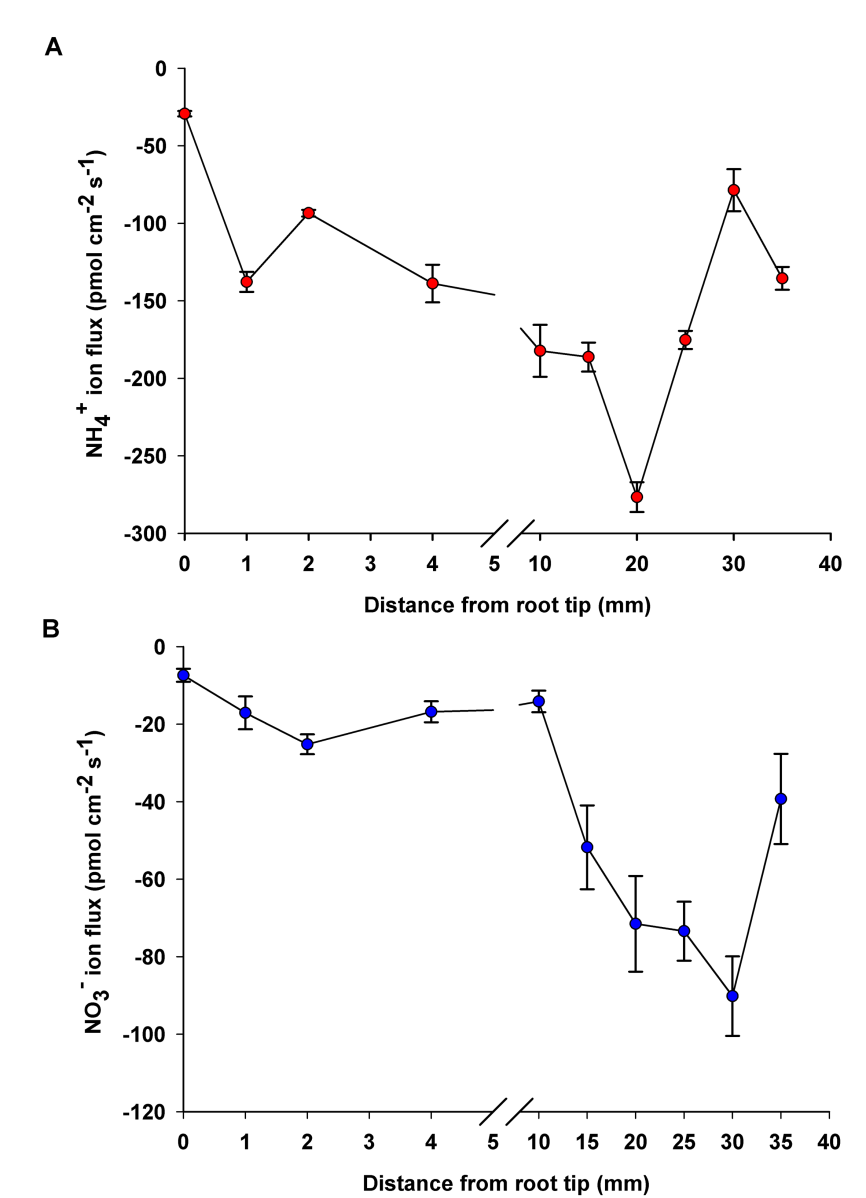


**Supplementary Figure S2 |** Net NH_4_^+^ fluxes **(A)** and NO_3_^-^ fluxes **(B)** along root tips of *B. campestris*. The data represent the mean ± SE (n=3). Net influxes are suggested by negative values, whereas net effluxes are indicated by positive values.

**Supplementary Table1 |** The primers were used in the study

| Primer name | Sequence (5′–3′) | Usage | |
| --- | --- | --- | --- |
| BcAMT1.2 | F:GCAGGTCGACTCTAGAATGGACATCGCAGCCACCACCT  R:ACGAGCTCGGTACCCGGGTCAAGCAGTTAAGGGCTGTGGT | Cloning *BcAMT1.2* into pCAMBIA3301 vector using *Xba* I and *Sma* I sites |  |
| pYES1.2 | F:CAGTGTGCTGGAATTCATGGACATCGCAGCCACCACCT  R:ATGCGGCCCTCTAGATCAAGCAGTTAAGGGCTGTGGT | Cloning *BcAMT1.2* into pYES2 vector using *EcoR* I and *Xba* I sites |  |
| pBI1.2 | F:CACGGGGGACTCTAGAATGGACATCGCAGCCACCACCT  R:TCCTTTACCCATCCCGGGAGCAGTTAAGGGCTGTGGT | Cloning *BcAMT1.2* into pBI121 vector using *Xba* I and *Sma* I sites |  |
| BcAMT1.2_pro_ | F:CCGGCGCGCCAAGCTTACGTCATCATACTTTATTACATG  R:GGGATCCGTCGACCTGCAGTAAGCAGGGGTAGATGCCTT | Cloning *BcAMT1.2_pro_* into pCAMBIA1391 vector using *Hind* III and *Pst* I sites |  |
| q-BcAMT1.1 | F: CTTCAACAAGATCCTCGTCAC  R: GTTATAGCTGCAAACCCTCC | qRT-PCR for the cDNA of *Brassica campestris* | |
| q-BcAMT1.2 | F: CTGTCTGGAATGTAGTGTCG  R: TCTCCATCTCCCCAAAGAGC |  |  |
| q-BcAMT1.3 | F: TCGGAGAAGGATGAGATGG  R: CGAGGAGGAGTAACAGAACG |  |  |
| q-BcAMT1.5 | F: AGTGCCACAATGGGAACAC  R: GAGCTGGAGGATCAGAACG |  |  |
| q-BcNRT1.1 | F: CCAACGACCTCGTCTCAT  R: CCTTCACTCCTCCAGTTCCT |  |  |
| q-BcNRT1.8 | F: CAGGAGATACAGGGAGGAGGA  R: TTTGGGTGAAGACGACCGAG |  |  |
| q-BcNRT2.1 | F: TCCAGTTAAAGGTACGGAGGAG  R: TAGCAGAGCGGCTACGAG |  |  |
| q-BcNRT3.1 | F: ATGGCGCAAGACGGAAAATG  R: GGTTTTGACAGTGGGGTCAT |  |  |
| q-BcNAXT1 | F: TTCTCACACCGCTCCAACAA  R: AGCACAGAGCTACATTCCCT |  |  |
| GAPDH | F: CAGGTTTGGAATTGTCGAGG  R: GAGCTGTGGAAGCACCTTTC |  |  |

| AtGLN1.1 | F: CAACCTTAACCTCTCAGACTCCACT  R: CAGCTGCAACATCAGGGTTGCTA | qRT-PCR for the cDNA of *Arabidopsis* |
| --- | --- | --- |
| AtGLN1.2 | F: TAACCTTGACATCTCAGACAACAGT  R: TCAGCAATAACATCAGGGTTAGCA |  |
| AtGLN2 | F: CCAACATGTCAGATGAGAGTGCC  R: CCAGGTGCTTGACCGGTACTCG |  |
| AtGDH2 | F: CCAACATGTCAGATGAGAGTGCC  R: CCAGGTGCTTGACCGGTACTCG |  |
| AtGLT1 | F: GGTCTTCCATGGGAACTGGG  R: TAGCAATTCCCACAGGGCAG |  |
| AtACTIN2 | F: TCGGTGGTTCCATTCTTGCT  R: GCTTTTTAAGCCTTTGATCTTGAGAG |  |
| Bar | F: ATGAGCCCAGAACGACGCC  R: TCAAATCTCGGTGACGGGCA | PCR for the transgenic *Arabidopsis* |

**Supplementary Table S2 |** Accession numbers of AMT proteins extracted from different databases and used for the phylogenetic analysis.

AMTs：

*Arabidopsis thaliana:*

AtAMT1.1 (NP_193087.1), AtAMT1.2 (NP_176658.1), AtAMT1.3 (NP_189073.1), AtAMT1.4 (NP_194599.1), AtAMT1.5 (NP_189072.1), AtAMT2 (NP_181363).

*Brassica rapa:*

BrAMT1.1 (XP_009124614), BrAMT1.2 (XP_009111404.1), BrAMT1.3 (XP_009102522.1), BrAMT1.4 (XP_009129394.1), BrAMT1.5 (XP_009135885.1), BrAMT2 (XP_009143404.1), BrAMT2like (XP_009141731.1).

*Oryza sativa*:

OsAMT1.1 (XP_015636241.1), OsAMT1.2 (XP_015623207.1), OsAMT1.3 (XP_015624850.1), OsAMT2.1 (XP_015639562.1), OsAMT2.2 ([XP_015643018.1](https://www.ncbi.nlm.nih.gov/protein/XP_015643018?report=genbank&log$=protalign&blast_rank=7&RID=CVE66BHU014)), OsAMT2.3 ([XP_015621584.1](https://www.ncbi.nlm.nih.gov/protein/XP_015621584?report=genbank&log$=protalign&blast_rank=8&RID=CVE66BHU014)), OsAMT3.1 (XP_015622013.1), OsAMT3.2 (XP_015630045.1), OsAMT3.3 (XP_015626434.1), OsAMT4.1 (Q10CV4.1).

*Populus trichocarpa*:

PtrAMT1.1 ([XP_002314518.2](https://www.ncbi.nlm.nih.gov/protein/566189592)), PtrAMT1.2 ([XP_002325790.1](https://www.ncbi.nlm.nih.gov/protein/224145861)), PtrAMT1.4 (XP_002301837.1), PtrAMT2 ([XP_002309151.1](https://www.ncbi.nlm.nih.gov/protein/224091028)), PtrAMT2.2 ([XP_002323600.2](https://www.ncbi.nlm.nih.gov/protein/566210003)), PtrAMT3.1 ([XP_002324271.3](https://www.ncbi.nlm.nih.gov/protein/1375915716)), PtrAMT4.1 (XM_002302048.1).


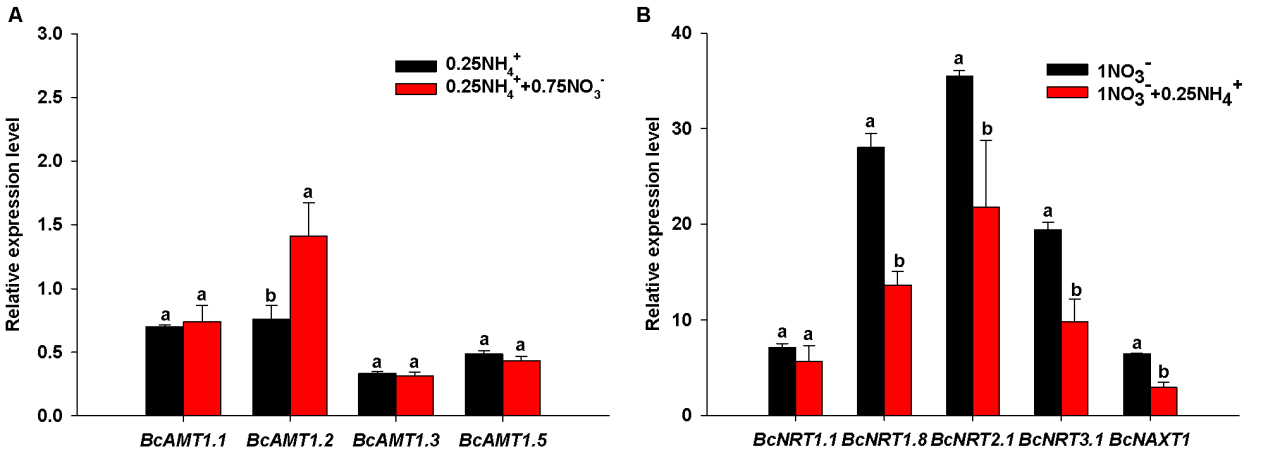
**FIGURE S3** | Effects of adding NO_3_^-^ on *AMTs* expression levels and adding NH_4_^+^ on *NRTs* expression levels. **(A)** *BcAMTs* expression after adding 0.75 mmol L^-1^ NO_3_^-^ in the nutrition of 0.25 mmol L^-1^ NH_4_^+^. **(B)** *BcNRTs* expression after adding 0.25 mmol L^-1^ NH_4_^+^ in the nutrition of 1 mmol L^-1^ NO_3_^-^. The data represent the mean ± SE (n=6). Significant differences (*P<0.05*) between treatments are indicated by different letters.


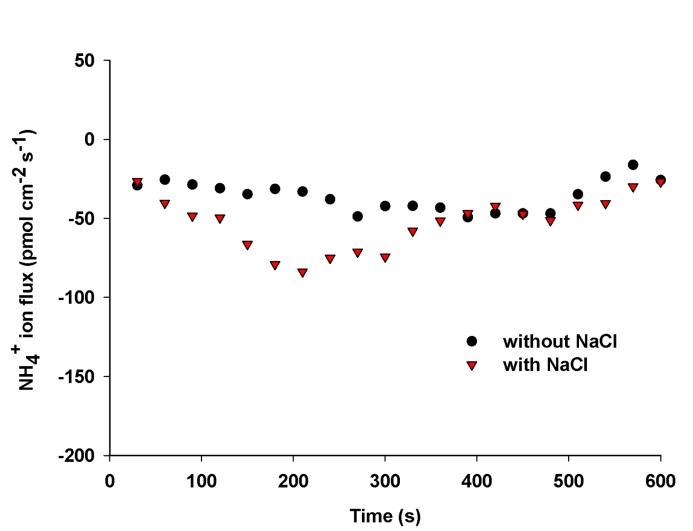


**Supplementary Figure S4 |** Effect of Na^+^ on NH_4_^+^ net fluxes on root surfaces of *B. campestris*


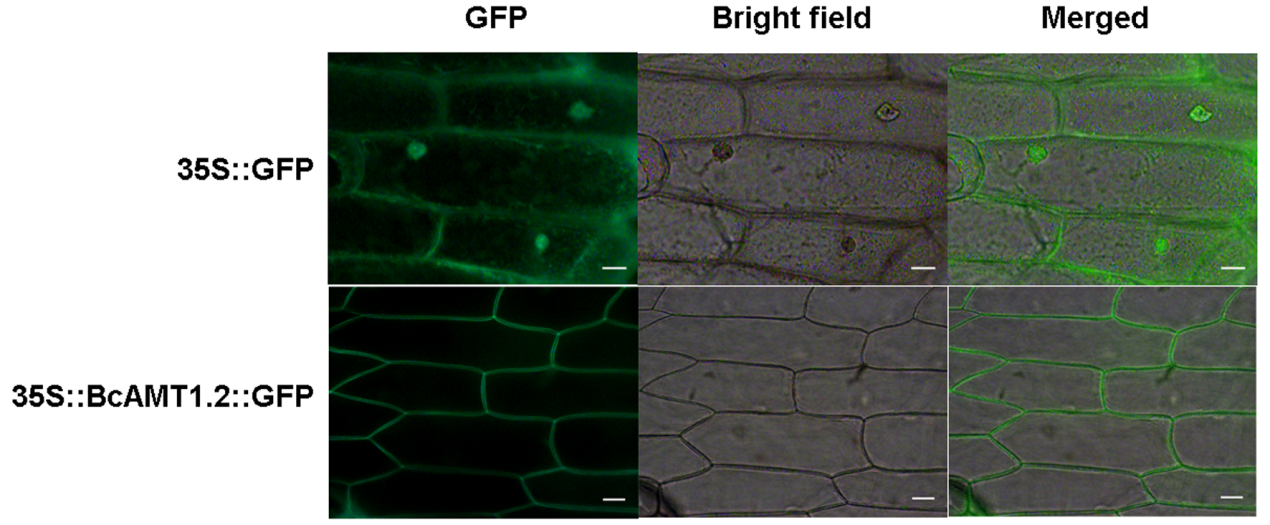


**Supplementary Figure S5 |** Subcellular localization of 35S::GFP and 35S::BcAMT1.2::GFP fusion proteins in onion epidermal cells. Bright field: image obtained by bright field microscopy; GFP: green fluorescence derived from GFP imaged by fluorescence confocal microscopy; Merged: overlay of the two images. Scale bar: 50 μm.


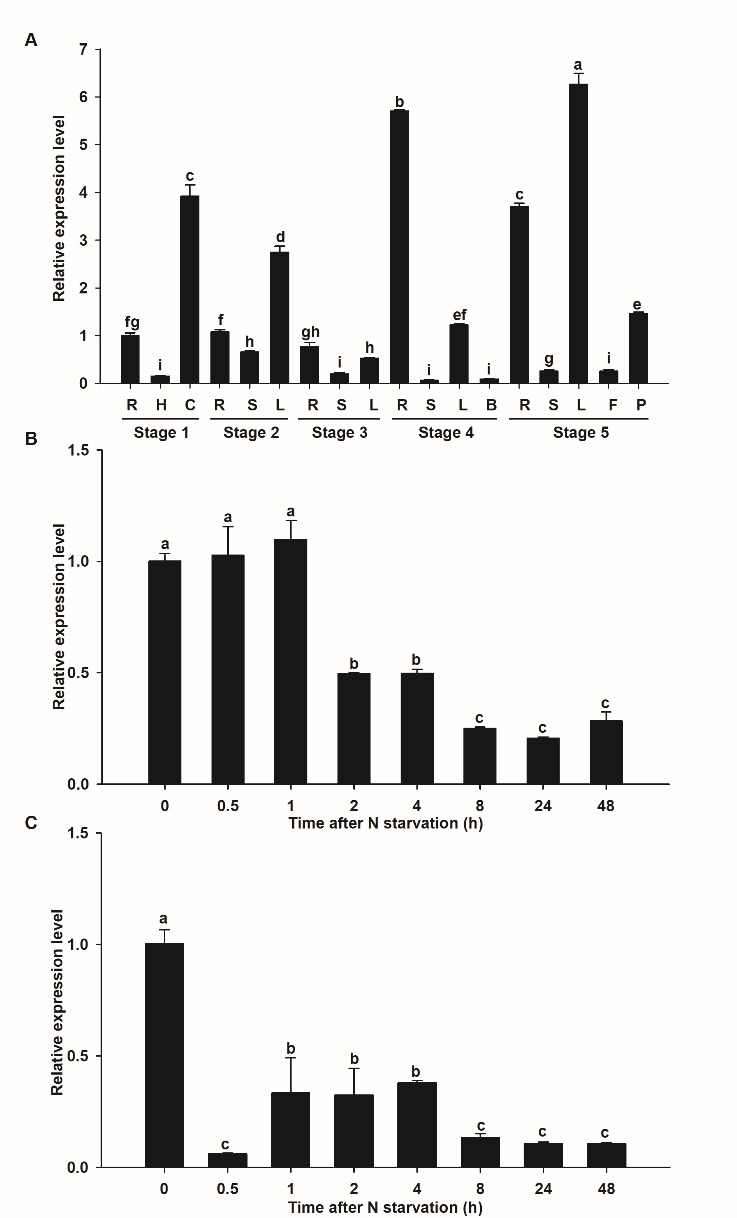


**Supplementary Figure S****6 |** *BcAMT1.2* expressions in different organs and under N starvation for different times of *B. campestris*. (**A)** *BcAMT1.2* expressions in different organs of *B. campestris* during five developmental stages. Stage1: at the cotyledon stage; stage 2: the three-leaf stage; stage 3: the six-leaf stage; stage 4: the stalk-growth stage; and stage 5: the flowering stage. R: root, H: hypocotyl, C: cotyledons, S: stem, L: leaf, B: flower bud, F: flower, and P: pod. **(****B)** Effect of N starvation on the expression of *BcAMT1.2* in *B. campestris* roots. (**C)** Effect of N starvation on the expression of *BcAMT1.2* in *B. campestris* leaves. The transcript levels of *BcAMT1.2* were normalized to the expression of glyceraldehyde-3-phosphate dehydrogenase gene. Each value represents the mean ± SE (n=3). Different lowercase letters indicate significant differences at *P<0.05*.
